# Supplementary material for: Molecular Recalibration of PD-1+ Antigen-Specific T Cells from Blood and Liver
Source: Mol Ther. 2018 Aug 16;26(11):2553–66. doi: 10.1016/j.ymthe.2018.08.013 (PMC6225092; doi:10.1016/j.ymthe.2018.08.013)

YMTHE, Volume *26*

## Supplemental Information

# Molecular Recalibration of PD-1+

# Antigen-Specific T Cells from Blood and Liver

**Itziar Otano, David Escors, Anna Schurich, Harsimran Singh, Francis Robertson, Brian R. Davidson, Giuseppe Fusai, Frederick A. Vargas, Zhi M.D. Tan, Jia Y.J. Aw, Navjyot Hansi, Patrick T.F. Kennedy, Shao-An Xue, Hans J. Stauss, Antonio Bertoletti, Andrea Pavesi, and Mala K. Maini**

Supplementary Figure 1

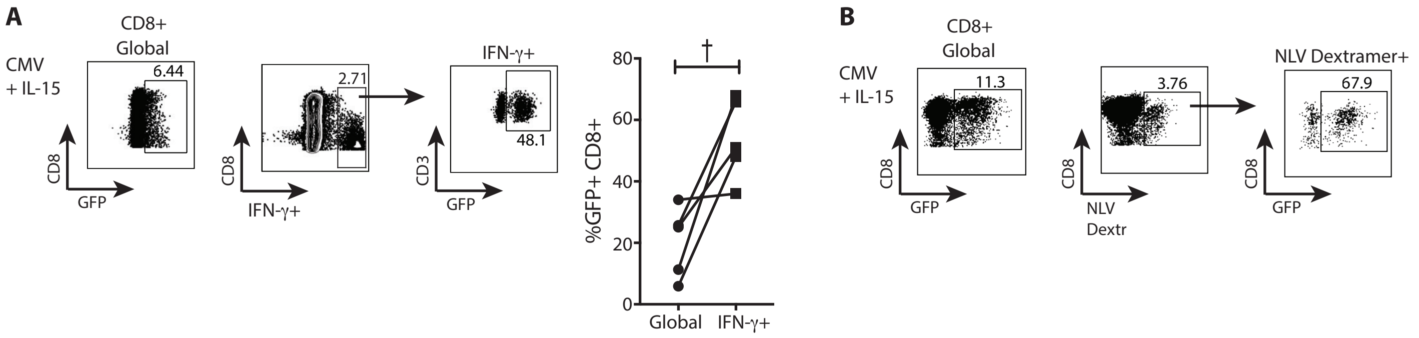

Supplementary Figure 2

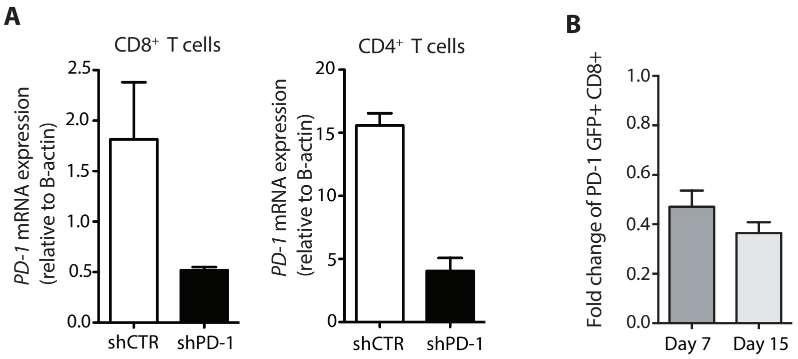

# Supplementary Figure 3

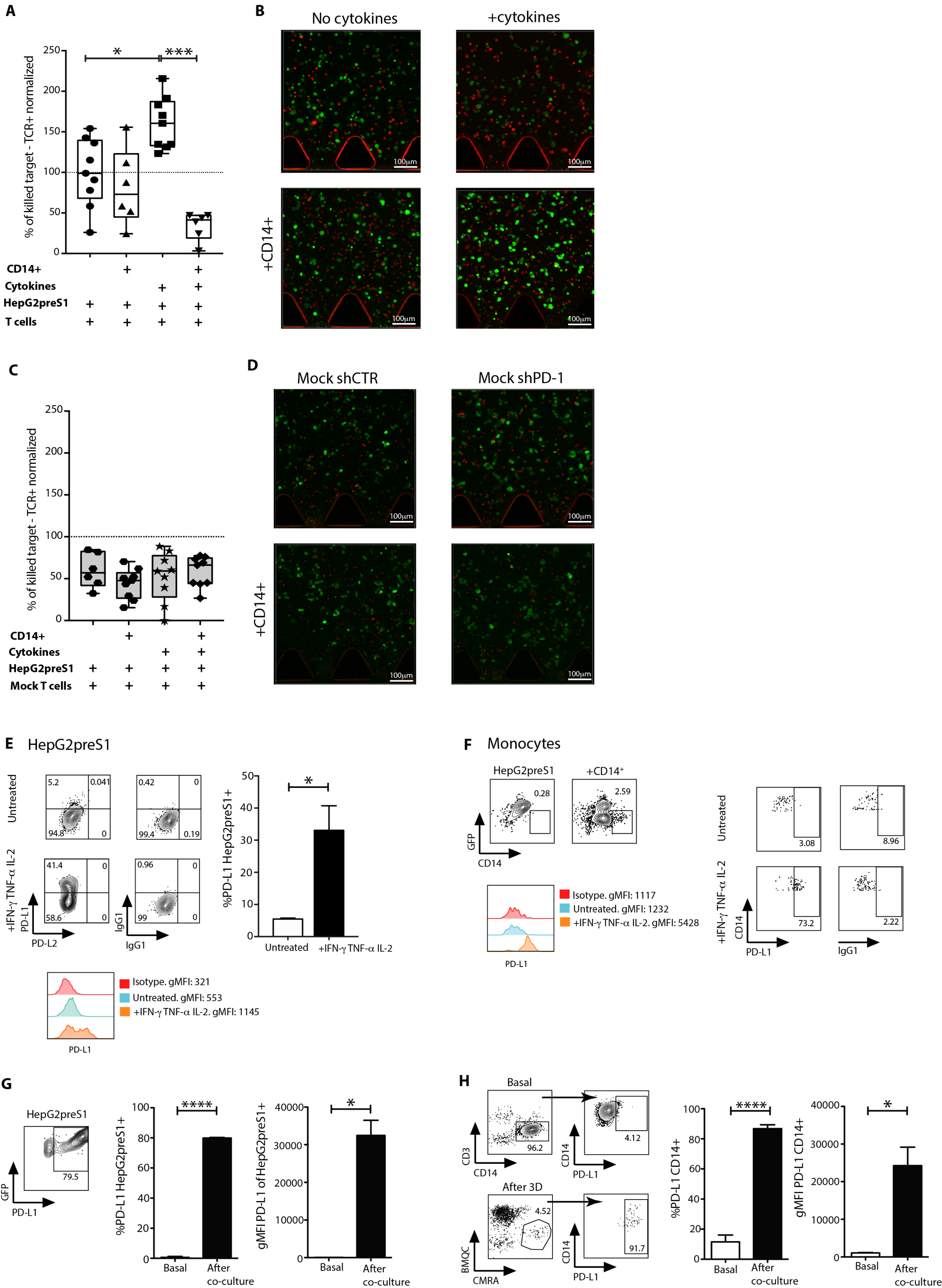

Supplementary Figure 4

A

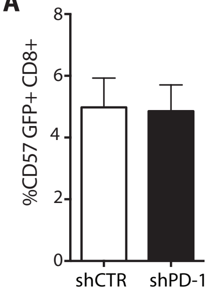

Supplement: Document S1. Figures S1–S4 [file mmc1.pdf]
