# Supplementary material for: Molecular Recalibration of PD-1+ Antigen-Specific T Cells from Blood and Liver
Source: Mol Ther. 2018 Aug 16;26(11):2553–66. doi: 10.1016/j.ymthe.2018.08.013 (PMC6225092; doi:10.1016/j.ymthe.2018.08.013)

Molecular Therapy
Original Article

Molecular Recalibration of PD-1+ Antigen-Specific T Cells from Blood and Liver

Itziar Otano,[1,4,5] David Escors,[1,6] Anna Schurich,[1,7] Harsimran Singh,[1] Francis Robertson,[2] Brian R. Davidson,[2] Giuseppe Fusai,[2] Frederick A. Vargas,[3] Zhi M.D. Tan,[5] Jia Y.J. Aw,[5] Navjyot Hansi,[8] Patrick T.F. Kennedy,[8] Shao-An Xue,[1,9] Hans J. Stauss,[1] Antonio Bertoletti,[5,10] Andrea Pavesi,[5] and Mala K. Maini[1]

[1]Division of Infection and Immunity, Institute of Immunity and Transplantation, UCL, London, UK; [2]Department of Surgery and Interventional Science, UCL, London, UK; [3]Institute of Cancer Research, UCL, London, UK; [4]Division of Immunity and Immunotherapy, Centre for Applied Medical Research, Pamplona, Spain; [5]Institute of Molecular and Cell Biology, Agency for Science, Technology and Research, Singapore, Singapore; [6]Navarrabiomed-Biomedical Research Centre, IdiSNA, Pamplona, Spain; [7]School of Immunology and Microbial Sciences, King's College London, London, UK; [8]Centre for Immunobiology, Blizard Institute, Bart's and the London School of Medicine and Dentistry, QMUL, London, UK; [9]Genetic Engineering Laboratory, School of Biological and Environmental Engineering, Xi'an University, Xi'an, China; [10]Emerging Infectious Diseases Program, Duke-NUS Graduate Medical School, Singapore, Singapore

Checkpoint inhibitors and adoptive cell therapy provide promising options for treating solid cancers such as HBV-related HCC, but they have limitations. We tested the potential to combine advantages of each approach, genetically reprogramming T cells specific for viral tumor antigens to overcome exhaustion by down-modulating the co-inhibitory receptor PD-1. We developed a novel lentiviral transduction protocol to achieve preferential targeting of endogenous or TCR-redirected, antigen-specific CD8 T cells for shRNA knockdown of PD-1 and tested functional consequences for antitumor immunity. Antigen-specific and intrahepatic CD8 T cells transduced with lentiviral (LV)-shPD-1 consistently had a marked reduction in PD-1 compared to those transduced with a control lentiviral vector. PD-1 knockdown of human T cells rescued antitumor effector function and promoted killing of hepatoma cells in a 3D microdevice recapitulating the pro-inflammatory PD-L1^{hi} liver microenvironment. However, upon repetitive stimulation, PD-1 knockdown drove T cell senescence and induction of other co-inhibitory pathways. We provide the proof of principle that T cells with endogenous or genetically engineered specificity for HBV-associated HCC viral antigens can be targeted for functional genetic editing. We show that PD-1 knockdown enhances immediate tumor killing but is limited by compensatory engagement of alternative co-inhibitory and senescence program upon repetitive stimulation.

INTRODUCTION

CD8 T cells are critical for immune control of persistent viral infections and cancer. In both these settings, T cell efficacy is limited by low frequencies and functional defects, which are promoted by chronic exposure to antigen and/or inflammation, a scenario termed exhaustion.[1] A cardinal feature of exhausted T cells is excessive negative co-regulation through multiple receptors, with PD-1 constituting a pivotal co-inhibitory receptor in these settings.[1,2] The capacity to restore immune control of infection and cancer by *in vivo* PD-1 blockade was first demonstrated in animal studies and more recently exemplified by ground-breaking results in patients with melanoma and other solid tumors.[1–3] Although the use of checkpoint inhibitors such as PD-1 blocking antibodies is revolutionizing cancer therapy for a proportion of patients, there remain significant limitations inherent to this approach. A therapeutic response to antibody-mediated checkpoint blockade requires the tumor to have a relatively high mutation burden and a pre-existing lymphocytic infiltrate.[4–6] The use of blocking monoclonal antibodies means that effects are of limited duration and require repeated dosing, with its associated problems. Cells expressing PD-1 will potentially be affected, resulting in the unleashing of bystander and autoreactive T cell specificities and a substantial risk of autoimmune disease.[7] Regulatory populations such as Tregs can also express high levels of PD-1, so PD-1 blockade can expand regulatory T cells (Tregs), which will tend to counteract the boosting of effector T cells.[8]

A potentially elegant solution for these limitations is to attempt selective genetic knockdown of PD-1 on T cells of the desired specificity. To date, genetic engineering of T cells has targeted mitogen-activated bulk T cells, rather than those of a particular specificity. This could result in genetic modification of irrelevant and potentially harmful subsets and specificities, as in the use of blocking antibodies. In addition, inefficient transduction rates may mean that low-frequency, antigen-specific T cells are not targeted. In this study, we have investigated two approaches to achieving selective knockdown of PD-1 on antigen-specific T cells. First, we have developed a protocol to focus

Received 15 May 2018; accepted 10 August 2018;
https://doi.org/10.1016/j.ymthe.2018.08.013.

Correspondence: Andrea Pavesi, Institute of Molecular and Cell Biology, Agency for Science, Technology and Research, 61 Biopolis Drive, #03-06 Proteos Building, Singapore 138673, Singapore.
E-mail: andreap@imcb.a-star.edu.sg
Correspondence: Mala K. Maini, Division of Infection and Immunity, Institute of Immunity and Transplantation, UCL, Rayne Building, 5 University Street, London WC1E 6JF, UK.
E-mail: m.maini@ucl.ac.uk

This is an open access article under the CC BY license (http://creativecommons.org/licenses/by/4.0/).

lentiviral transduction of short hairpin RNAs (shRNAs) on peptide-specific T cells. Second, we have combined PD-1 knockdown with TCR gene transfer to confer antigen specificity.

As a proof of principle for these novel approaches, we have used commonly targeted human leukocyte antigen (HLA)-A2-restricted epitopes within HBV proteins. These targets are of major clinical relevance in the development of T cell therapy for chronic hepatitis B (CHB) and HBV-related hepatocellular carcinoma (HCC).[9] CHB and HCC are characterized by very low-frequency, antigen-specific CD8 T cell responses expressing high levels of PD-1.[10–13] HBV-related HCC often has integrated HBV DNA and can express HBV antigens, rendering it susceptible to killing by HBV-specific T cells.[9] We contributed to the first-in-man use of TCR-redirected T cells to treat a patient with HBsAg-expressing HCC metastases.[14] This case supported the feasibility and safety of using HBV-specific adoptive T cell therapy in HCC. However, such autologous TCR gene-transferred T cells remain susceptible to inactivation through their expression of PD-1 in analogous settings.[15–18]

In the current study, we therefore edit PD-1 expression to favor the survival of either endogenous or TCR-redirected, tumor-specific T cells within the PD-L1^{hi} environment characteristic of the liver and tumors.[2,19,20] We show that it is feasible to target endogenous and TCR-redirected, virus-specific T cells with a lentivirus vector carrying shRNA to knock down PD-1. In light of accumulating evidence that tissue and tumor-resident T cells harbor unique adaptations to their niche,[21,22] we also test the feasibility to carry out genetic modification of liver-extracted T cells. We demonstrate that PD-1 knockdown on HBV-associated, HCC antigen-specific T cells boosts their effector function and capacity to kill tumor cells in a PD-L1^{hi} 3D microfluidic system. However, we find that PD-1 knockdown also limits the capacity of human CD8 T cells to withstand repetitive TCR stimulation and drives compensatory engagement of an alternative co-inhibitory and senescence program.

RESULTS

Preferential Targeting of Virus-Specific T Cells for Genetic Manipulation

T cells directed against an antigenic target constitute a small fraction of the global population. We investigated whether it was possible to direct lentiviral transduction toward these small, endogenous T cell populations of a particular specificity and thus achieve their selective genetic manipulation while sparing the remaining T cells. Because lentiviral transduction favors T cells transitioning into the G1b phase of the cell cycle,[23] we replaced the prototype polyclonal T cell activation protocol with stimulation using peptides representing viral epitopes.

We initially tested the possibility of targeting cytomegalovirus (CMV)-specific CD8 T cells, because these are an important therapeutic target and a useful test population, with a highly conserved immunodominant HLA-A2-restricted response that is usually detectable in the circulation at much higher frequencies than HBV-specific CD8 T cells. Pre-stimulation of isolated CD8 T cells with CMV peptide NLV for 24 hr was successful in focusing vesicular stomatitis virus G-protein (VSV-g) pseudotyped lentiviral transduction on the responding (interferon γ+ [IFN-γ+]) population (Figure 1A). Up to 67.6% of the CMV-specific CD8 T cells were transduced with a GFP-expressing control lentivirus, whereas only a minimal proportion of the global CD8 T cells were transduced (0.5% to 4.4% GFP+) (Figure 1A). Addition of the cytokine IL-15 tended to increase the expansion of virus-specific CD8 T cells (as previously described[24]) but at the expense of some increase in background transduction of global CD8 (Figures 1A and S1A). Staining with an HLA-A2/NLV peptide dextramer confirmed preferential transduction of more than 60% of CMV-specific CD8 T cells (Figures 1B and S1B).

We then tested the feasibility of specifically transducing the small populations of HBV-specific CD8 T cells circulating in patients with chronic HBV infection using the same protocol. As seen with CD8 T cells directed against CMV, there was enrichment for transduction of HBV-specific CD8 T cells compared to the global fraction (Figure 1C). The percentage of transduced (GFP+) HBV-specific CD8 T cells tended to increase in the presence of interleukin-15 (IL-15), reaching up to 63.2% of IFN-γ-expressing cells. Furthermore, efficient transduction of HBV-specific CD8 T cells could be achieved stimulating whole peripheral blood mononuclear cell (PBMC) rather than purified CD8 T cells (Figure 1D). These experiments confirmed that it was possible to target lentiviral transduction toward CD8 T cells specific for HBV antigens commonly expressed by HCC. This was consistently achieved for the more robust responses directed against CMV but was also feasible to a variable degree for low-frequency, HBV-specific T cells.

PD-1 Knockdown on Human Antigen-Specific T Cells

Having developed a protocol for targeted transduction of antigen-specific T cells, we then tested the potential to introduce shRNAs for their molecular modification. We focused on knockdown of the co-inhibitory receptor PD-1, an important therapeutic target in chronic viral infection and cancer. We first tested the capacity of lentiviral vectors carrying GFP and either shRNA against PD-1 (lentiviral [LV]-shPD-1) or control shRNA (LV-shCTR) to knock down PD-1 on global human CD8 T cells. shPD-1 (Sigma) was designed to maximize efficient knockdown of only the target gene and was not predicted to target any off-target transcripts on screening using GESS or SPICE. The percentage of transduced (GFP+) CD8 T cells expressing PD-1 was significantly reduced by LV-shPD-1 compared to LV-shCTR (flow cytometry plots and summary data, Figure 2A) (qPCR, Figure S2A). Consistent with the capacity of lentiviral-transduced shRNA to achieve long-term silencing of genes, stable PD-1 knockdown was maintained over 2 weeks in culture (Figure S2B).

We next tested shRNA knockdown of PD-1 using peptide-stimulated lentiviral transduction of endogenous virus-specific T cells. Peptide-loaded T2 cells were used to restimulate genetically manipulated CD8 T cells. CMV-specific CD8 T cells, identified by IFN-γ staining (Figure 2B) or HLA-A2/NLV dextramer staining (Figure 2C)

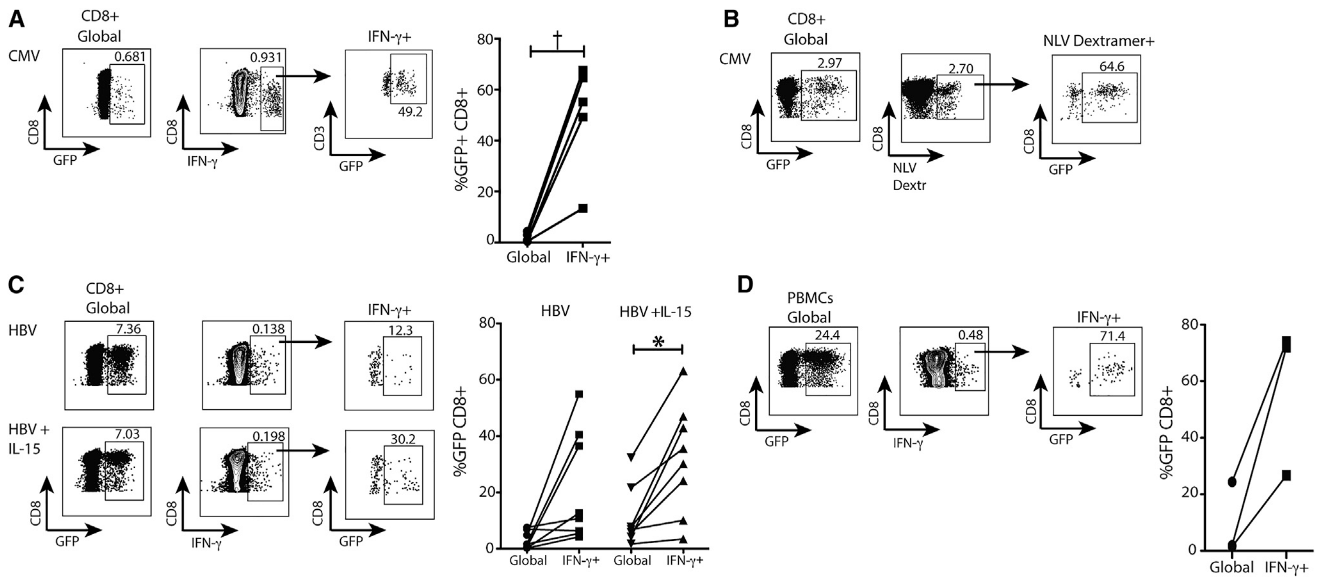

Figure 1. Selective Lentiviral Transduction of Virus-Specific CD8 T Cells

Isolated CD8 T cells were stimulated with HLA-A2-restricted viral peptides ± IL-15 for 24 hr. Cells were then transduced with lentiviral particles expressing GFP ± a control shRNA (LV-shCTR) and cultured for 7 days. (A and B) Representative examples of GFP expression in global CD8 T cells and in CMV-specific CD8 T cells gated on intracellular IFN-γ+ (A) and HLA-A2/NLV-dextramer+ (B) CD8 T cells upon restimulation with NLV-loaded T2 cells. Summary data comparing GFP expression in global and CMV-specific CD8 T cells (n = 4 healthy control [HC] and n = 1 CHB). Data were analyzed with paired t test. (C) GFP expression in global CD8 T cells and in HBV-specific CD8 T cells gated on intracellular IFN-γ+CD8 T cells upon restimulation with HBV/HLA-A2+ peptide-loaded T2 cells. Non-peptide-loaded T2 cells were used to restimulate T cells as a control for non-specific background IFN-γ staining, which was subtracted from that obtained using NLV or HBV peptide-loaded T2 cells. Summary data comparing GFP expression in global and HBV-specific CD8 T cells (n = 8, CHB). Data were analyzed with one-way ANOVA and Dunn's multiple comparison test. (D) PBMCs from CHB patients (n = 3) were lentivirally transduced (without purifying CD8 T cells) after 24 hr of stimulation, with overlapping peptides spanning the HBV core plus IL-15, and cultured for 10 days. Representative dot plots of GFP expression in global and in IFN-γ + CD8 T cells after restimulation with HBV core-overlapping peptides and summary data. In (A)–(C), CD8+ T cells were pre-gated on live/CD19−/CD3+, whereas in (D), CD8 T cells were pre-gated on live+/CD3+/CD4−. Significant differences are †$p < 0.1$ and *$p < 0.05$.

following 24 hr stimulation with peptide and IL-15, were transduced with lentiviral vectors. A high proportion of transduced GFP+ cells expressed PD-1 with LV-shCTR (up to 91.8%), whereas PD-1 expression was almost abrogated using LV-shPD-1 (Figures 2B and 2C). Similarly, PD-1 expression was partially abrogated on low-frequency HBV-specific CD8 T cells (Figure 2D).

PD-1 Knockdown of TCR-Redirected Peripheral and Liver-Infiltrating T Cells

To overcome the limitation of the very low-frequency of HBV-specific CD8 T cells in patients with CHB and HBV-related HCC, it is possible to reengineer T cells able to recognize HBV epitopes by TCR gene transfer.[14,25] However, adoptively transferred T cells have been shown to remain susceptible to PD-1-mediated exhaustion *in vivo*.[15–17] We therefore tested the possibility of dual genetic manipulation of human CD8 T cells to render them both specific for HBV and resistant to PD-1 inhibition. T cells engineered to express a TCR specific for an HLA-A2-restricted epitope from HBV core or envelope (core$_{18–27}$ or env$_{183-91}$, using a retroviral vector as described previously[25]) were identified and underwent fluorescence-activated cell sorting (FACS) based on their expression of the murine portion of the Cβ chain (CβmTCR) (Figure 3A). Purified TCR-redirected T cells were then transduced with lentiviral vectors carrying shCTR. This confirmed the feasibility of dual transduction; cells remained viable and functional, producing high levels of IFN-γ and tumor necrosis factor alpha (TNF-α) upon stimulation with the T2 cell line loaded with cognate peptide (Figure 3B). We then tested whether we were able to reduce PD-1 expression on TCR-transduced T cells following a second transduction using LV-shPD-1. T cells redirected against either core$_{18–27}$ or env$_{183-91}$, identified by staining for CβmTCR or an HLA-A2/env$_{183-91}$ dextramer, showed reduced PD-1 following a second transduction with LV-shPD-1 compared to LV-shCTR (Figures 3C and 3D).

The literature has highlighted the unique properties of tissue-resident immune cells,[21,22] which could potentially be exploited to promote retention and survival of genetically modified T cells at the site of interest. Tumor-infiltrating lymphocytes have previously been extracted and expanded for adoptive therapy to enrich for T cells with the correct specificity and homing characteristics.[26] We therefore tested whether it was possible to genetically modify liver-resident T cells, which are adapted to persist at the site of HBV and HCC disease. For this purpose, intrahepatic T cells were extracted from biopsies of HBV-infected livers, healthy liver perfusates, or margins of liver cancer resections; stimulated with a cocktail of cytokines known to promote liver residence;[21] and transduced with LV-shCTR.

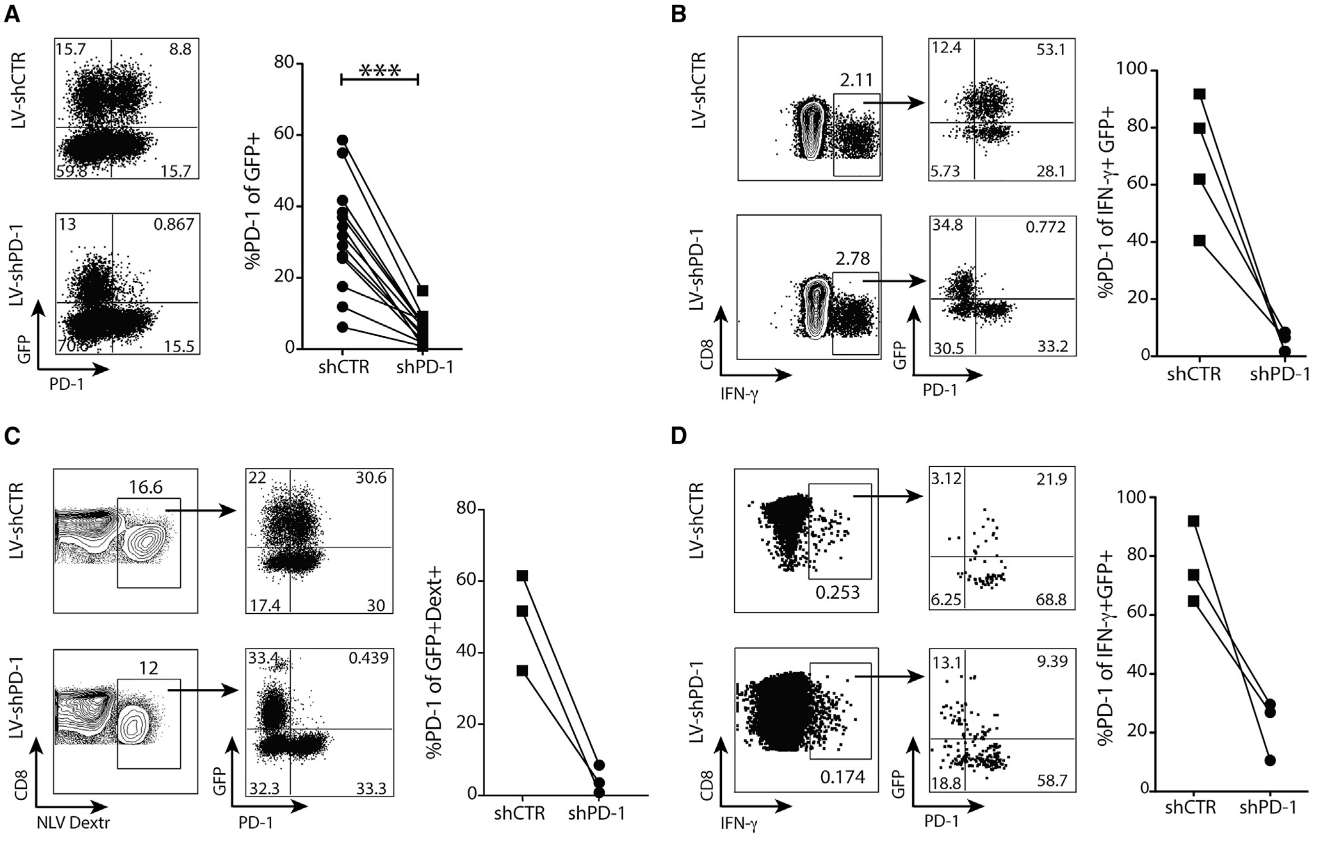

Figure 2. PD-1 Knockdown on Virus-Specific CD8 T Cells

PD-1 levels were analyzed in antigen-specific CD8 T cells after peptide-stimulated transduction with a lentiviral vector expressing a short hairpin against PD-1 (LV-shPD-1) or a control shRNA (LV-shCTR). (A) Representative FACS dot plot of PD-1 expression on global GFP+CD8 T cells and summary data (n = 4 HC and n = 9 CHB) (p = 0.0002, Wilcoxon rank-sum test). Seven days after lentiviral transduction, virus-specific CD8 T cells were identified by IFN-γ production and HLA-A2-peptide dextramer staining. (B and C) Lentiviral transduced CMV-specific CD8 T cells were analyzed for PD-1 expression on IFN-γ+ (B) and HLA-A2/NLV-dextramer+ (C) CD8 T cells after stimulation with NLV peptide-loaded T2 cells. Summary data of GFP+ CMV-specific CD8 T cells (n = 2 HC and n = 2 CHB for IFN-γ+ responses and n = 1 HC and n = 2 CHB for HLA-A2/NLV-dextramer+ staining). (D) PD-1 expression in HBV-specific CD8 T cells after lentiviral transduction analyzed on IFN-γ+ CD8 after stimulation with HBV/HLA-A2+ peptide-loaded T2 cells. Summary data of PD-1 levels on GFP+HBV-specific CD8 T cells from CHB patients (n = 3). Data were analyzed with Wilcoxon rank-sum test. CD8 T cells were pre-gated on live+/CD19−/CD3+. Significant differences are <0.1: ***p < 0.001. Any p value less than 0.001 was designated with three asterisks.

CD8 T cells extracted from these diverse liver samples could all be successfully transduced to achieve a mean expression of 36.8% GFP+ (Figure 3E). In some cases, we also transduced intrahepatic lymphocytes with LV-shPD-1, demonstrating partial knockdown of PD-1 (Figure 3F).

PD-1 Knockdown Increases CD8 T Cell Cytokine Release and Proliferation

To examine the potential of PD-1 knockdown to boost antitumor capacity of T cells, we first analyzed the impact on T cell effector function in standard 2D culture assays, in which some PD-L1 will be upregulated.[27] PBMC from patients with CHB were lentivirally transduced, expanded twice with α-CD3/CD28 antibodies for 15 days, and restimulated with α-CD3/CD28 antibodies. IFN-γ-producing and IFN-γTNF-α dual-producing CD8 T cells were significantly increased following repetitive stimulation of those populations transduced with LV-shPD-1 compared to LV-shCTR (Figures 4A and 4B). Similarly, CD8 T cells with PD-1 knockdown displayed enhanced expression of the key cytotoxic mediator granzyme B expression and of Ki67, a marker of cell proliferation, compared to control CD8 T cells (Figures 4C and 4D). PD-1 knockdown resulted in an initial upregulation of T-bet (Figures 4E and 4F), with the degree of induction correlating positively with IFN-γ production (Figure 4G).

Enhanced Antitumor Response in a 3D Model of the PD-L1^{hi} Liver Microenvironment

As demonstrated in our previous work,[28] classical 2D *in vitro* assays have limitations in evaluating the killing efficiency of engineered T cells. Therefore, to examine the antitumor potential of HBV-redirected, PD-1 knockdown engineered T cells, we used an innovative 3D microfluidic model, in which hepatoma cells expressing HBV preS1 protein covalently linked to GFP are seeded in a collagen gel in the central region of the device and T cells are injected into an

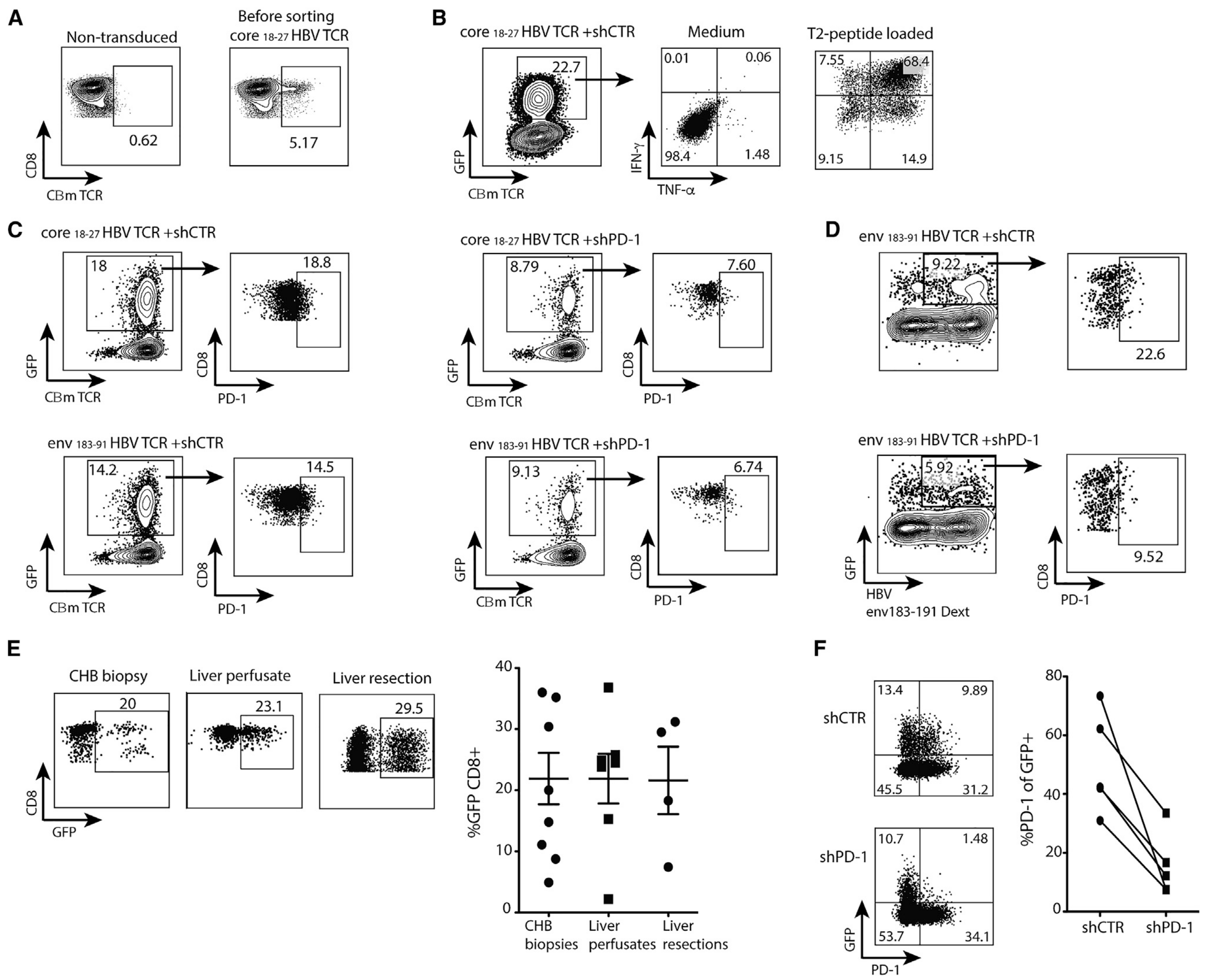

Figure 3. PD-1 Knockdown on HBV TCR-Redirected CD8 T Cells from the Intrahepatic and Peripheral Compartment

(A) FACS dot plots showing non-transduced and HBV core$_{18–27}$TCR CD8 T cells from a healthy donor before sorting. TCR expression was analyzed by the expression of murine Cβ region (CβmTCR). (B) Sorted TCR-redirected CD8 T cells were activated and transduced with LV-shCTR. After 7 days, the frequency of IFN-γ-TNF-α levels gated on GFP+CβmTCR+ was measured after stimulation with HBc$_{18–27}$ peptide-loaded T2 cells. (C) Representative FACS dot plots of PD-1 expression on GFP+CβmTCR+ CD8 T cells from a healthy donor after transduction of HBV core$_{18–27}$/env$_{182-191}$TCR with LV-shCTR or LV-shPD-1. (D) PD-1 levels in GFP+ HBV env$_{183-191}$TCR-redirected CD8 T cells gated on dextramer+ CD8 cells. Intrahepatic lymphocytes were activated for 24 hr with a cocktail of cytokines and transduced with LV-shCTR. (E) Representative FACS dot plots and summary data of the frequency of intrahepatic GFP+ CD8 T cells. (F) Representative FACS dot plots and summary data of PD-1 expression on intrahepatic CD8 T cells transduced with LV-shCTR or LV-shPD-1 (n = 5). Data were analyzed with Wilcoxon rank-sum test. CD8 T cells were pre-gated on live+/CD3+/CD4−, and in the case of restimulation with peptide-loaded T2 cells or dextramer staining, CD19-positive cells were excluded.

adjacent medium channel.[28] PBMCs from four healthy donors were transduced with LV-shCTR or LV-shPD-1 and expanded for 15 days before env$_{183–191}$TCR mRNA electroporation. Following flow cytometric confirmation of efficient lentiviral transduction and mRNA electroporation (55.7% to 73.9% HBV-TCR^{+}CD3), cells were injected into the 3D microfluidic system (Figure 5A).

We have shown that the pro-inflammatory cytokines IFN-γ, TNF-α, and IL-2 enhance the capacity of TCR-transfected T cells to lyse tumor target cells;[28] by contrast, the addition of autologous monocytes mimics the PD-L1^{hi} environment of the liver.[29] We therefore recapitulated these conditions in the microfluidic system to evaluate the benefit of PD-1 knockdown. First, we confirmed that target cell killing by env$_{183–191}$TCR-redirected T cells was increased in the presence of cytokines and inhibited when autologous monocytes were embedded in the collagen gel (Figures S3A and S3B), whereas mock-TCR-transduced T cells showed only basal tumor death (Figures S3C and S3D). PD-L1 was upregulated both on target tumor cells and on CD14+

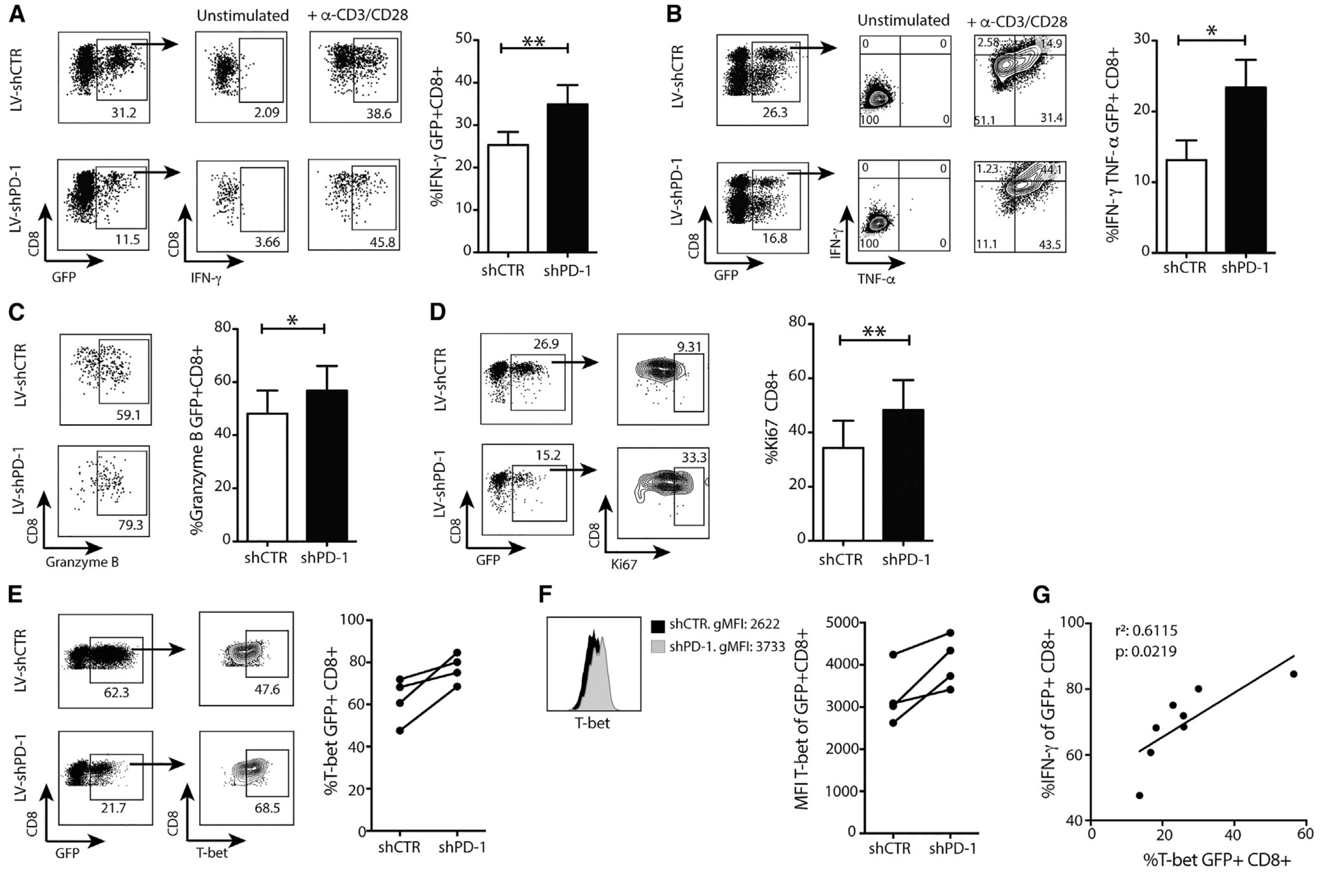

Figure 4. Functional Characterization of CD8 T Cells with PD-1 Knockdown

Activated PBMCs from CHB patients were transduced with LV-shCTR or LV-shPD-1 and then expanded with α-CD3/CD28 antibodies for 15 days. CD8 T cells responses were then analyzed after overnight α-CD3/CD28 stimulation. (A–D) Representative FACS dot plots and summary data showing the production of IFN-γ (A), IFN-γ-TNF-α (B), granzyme B (C) (n = 8, CHB), and Ki67 (D) (n = 8, CHB) from GFP+ CD8 T cells transduced with LV-shCTR or LV-shPD-1. (E and F) T-bet expression (E) and geometric mean fluorescent intensity (gMFI) (F) (n = 4, CHB) from GFP+ CD8+ T cells transduced with LV-shCTR or LV-shPD-1. (G) T-bet expressing according to the IFN-γ production (n = 8, CHB). CD8 T cells were pre-gated on live+/CD3+/CD4− and, in the case of (C), on GFP+. (A)–(F) were analyzed with Wilcoxon rank-sum test; (G) was analyzed with Pearson product-moment correlation coefficient. Significant differences are <0.1: *p < 0.05 and **p < 0.01. Error bars indicate SEM.

monocytes embedded in the 3D device collagen gel after 48 hr of culture with pro-inflammatory cytokines (Figures S3E and S3F) and even more strikingly after the addition of antigen-specific T cells (Figure S3G).

By labeling the TCR-redirected T cells with a fluorescent dye, we were able to quantify their invasion into the hydrogel, observing that this was increased by the presence of monocytes, but not by PD-1 knockdown (Figure 5B). The recovery of TCR-redirected T cells after co-culture and extraction from the microfluidic system was likewise not affected by PD-1 knockdown (Figure 5C). Target killing was assessed by the quantification of live (GFP+) or dead (DRAQ7-dye+) target cells after overnight co-culture with TCR-redirected T cells (Figures 5D and 5E). PD-1 knockdown significantly increased the efficiency of tumor cell killing with TCR-redirected T cells from 2 donors, but not from the other 2 donors. With the addition of PD-L1^{hi} monocytes, the effect of PD-1 knockdown on augmenting T cell antitumor efficacy became more consistent (Figures 5D and 5E).

PD-1 Knockdown Drives Compensatory Pathways to Limit the Persistence of Functional CD8 T Cells

The rationale to block the PD-1 co-inhibitory signal is based on its prominent role in settings of persistent antigenic stimulation and resulting T cell exhaustion. However, *in vivo* blockade and knockdown studies in animal models and patients have revealed additional complexity; PD-1 is just one of multiple non-redundant layers of potential T cell co-inhibitors[30] and may represent an adaptation that facilitates long-term lymphocyte survival by curtailing excessive stimulation.[21,31–34] Therefore, we investigated potential negative consequences of PD-1 genetic knockdown in human T cells from patients with CHB exposed to further repetitive antigenic stimulation *in vitro*. In this setting, PD-1 induced by persistent viral infection *in vivo* would be expected to be further upregulated by *in vitro* restimulation

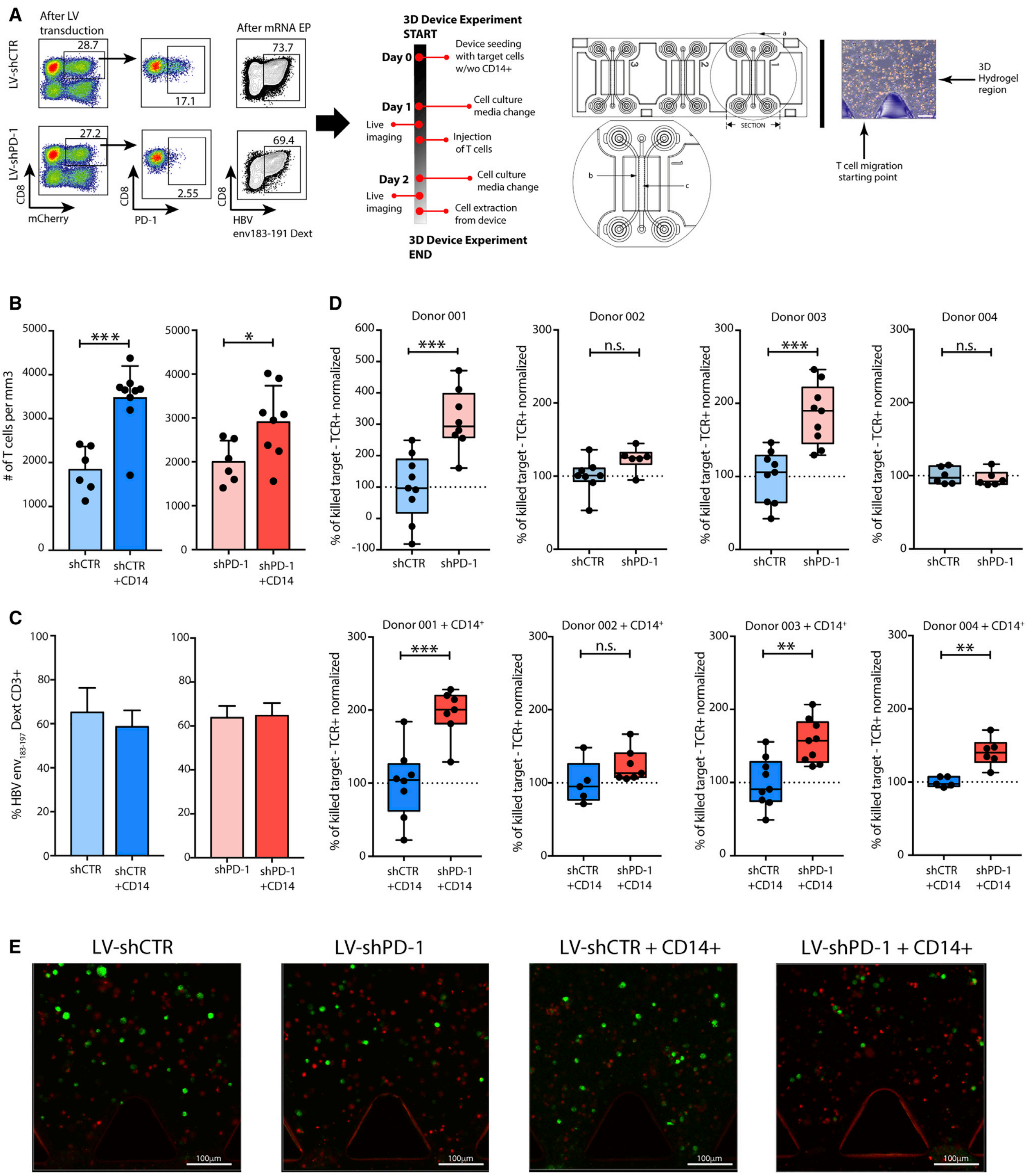

Figure 5. PD-1 Knockdown Boosts Antitumor Efficacy in PD-L1^{hi} 3D HCC Cultures

(A) CD8+ T cells were isolated from healthy donors, transduced with LV-shCTR or LV-shPD-1, and expanded for 15 days. Representative flow cytometry data demonstrate PD-1 knockdown efficiency and HBV-env T cell specificity after mRNA electroporation. A schematic of the DAX-1 3D microfluidic chip experimental timeline and design is shown, where a represents the zoomed section, b represents the liquid channel region, and c represents the hydrogel region. (B) The number of T cells (+/− PD-1

(legend continued on next page)

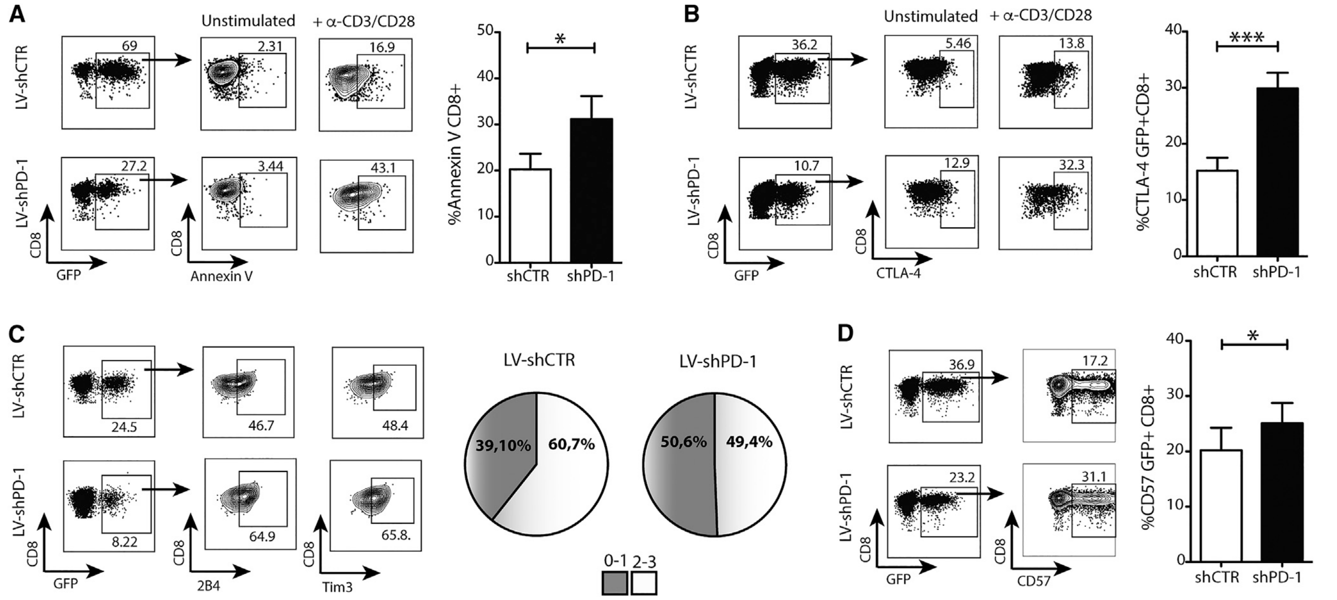

Figure 6. PD-1 Knockdown Drives Alternative Co-inhibitory and Senescence Program

Activated PBMCs from patients with CHB were transduced with LV-shCTR or LV-shPD-1 and then expanded with α-CD3/CD28 antibodies for 15 days. (A and B) Staining and summary data of annexin V (A) (n = 8, CHB) and CTLA-4 (B) (n = 13, CHB) on GFP+ CD8 T cells transduced with LV-shCTR or LV-shPD-1. (C) Example FACS plots of 2B4 and Tim-3 and Boolean gating analysis of the simultaneous expression of multiple inhibitory receptors (2B4, CTLA-4, and Tim-3) on GFP+ CD8 T cells transduced with LV-shCTR or LV-shPD-1 (n = 5, CHB). Pie charts display the percentage of individual populations grouped according to 0–1 and 2–3 co-expressed inhibitory receptors. (D) Expression and summary data of CD57 in GFP+ CD8 T cells (n = 13, CHB). All data were analyzed with Wilcoxon rank-sum test. CD8 T cells were pre-gated on live+/CD3+/CD4−. Significant differences are <0.1: *$p < 0.05$ and, ***$p < 0.001$. Any p value less than 0.001 was designated with three asterisks. Error bars indicate SEM.

in its role as a homeostatic regulator to curtail acute immune activation. Consistent with PD-1 providing a partially protective role, repetitively stimulated CD8 T cells with PD-1 knockdown showed compensatory changes constraining their survival and function. The FACS plots in Figure 6A exemplify the increase in the apoptotic marker annexin V following repetitive stimulation of CD8 T cells transduced with LV-shPD-1 compared to LV-shCTR. This suggested that removal of PD-1 inhibition increased their susceptibility to activation-induced cell death (Figure 6A). We postulated that PD-1 knockdown in the presence of ongoing T cell stimulation might also result in a compensatory increase in alternative co-inhibitory pathways. In support of this, we found that the negative regulator CTLA-4 was expressed at significantly higher levels on CD8 T cells on which PD-1 had been knocked down (Figure 6B). Combined analysis of CTLA-4, Tim-3 and 2B4 revealed that PD-1 knockdown CD8 T cells were more likely to co-express 2 or more of these co-inhibitory molecules than T cells transduced with the control vector (Figure 6C). We also observed an accumulation of CD57, a marker of end-stage differentiation toward immune senescence, on LV-shPD-1 CD8 T cells following repetitive rather than single restimulation (Figures 6D and S4). Altogether, these data reveal that PD-1 disruption on human CD8 T cells promotes upregulation of alternative pathways driving terminal differentiation and premature cell death.

DISCUSSION

HCC is the second-leading cause of cancer deaths worldwide; better treatments are urgently needed. Most cases arise on a background of chronic cirrhotic liver disease, with organ function already compromised. This provides a precarious backdrop in which to test novel immune-boosting therapies such as checkpoint inhibitors. Moreover, checkpoints such as the PD-1 pathway are strongly expressed on intrahepatic T cells and play a central role in maintaining the uniquely immunotolerant state of the liver.[11,21,35,36] Although checkpoint blockade trials have begun in patients with HCC[37] and viral hepatitis,[38,39] a more specific approach is desirable. The pivotal role of PD-1 in maintaining peripheral tolerance[40] places patients

knockdown) invading the region of interest was evaluated by confocal microscopy in the presence or absence of autologous CD14+ cells in the hydrogel (n = 4). Data were analyzed with one-way ANOVA with Tukey's test. (C) Percentage of HBV/HLA-A2 dextramer+CD3+ T cells recovered from the hydrogel 15 hr after co-culture, measured by flow cytometry (n = 3). T cells were pre-gated on live+/CD14−/CD3+/CD4−. (D) Summary data of HBV-expressing HepG2 cell death by engineered T cells after co-culture in the presence (top row) or absence (bottom row) of CD14+ cells (paired t test). The percentage of killed target cells (HepG2-PreS1-GFP) was evaluated by activation of DRAQ7 staining and normalized to adjust for variable transduction of HBV TCR-expressing T cells between donors, such that the mean value for shCTR without monocytes was set to 100% (a dot represents the percentage of killed targets in a single field). (E) Representative confocal maximum intensity projections of the preceding conditions (HepG2-PreS1 in green, dead cell staining DRAQ7 in red). Scale bars, 100 μm. Numbers of baseline dead cells in each device on day 1 were subtracted to calculate T cell-induced death on day 2. Significant differences are <0.1: **$p < 0.01$ and ***$p < 0.001$. Any p value less than 0.001 was designated with three asterisks. Error bars indicate SEM.

receiving systemic blockade at risk of autoimmunity.[7,41] Strong expression of PD-1/PD-L1 within the tumor microenvironment may predict a favorable response to PD-1 blockade[41,42] but conversely increases the risk of unleashing local collateral tissue damage.

In this study, we have demonstrated the feasibility of targeting transduction toward low-frequency, endogenous antigen-specific or intrahepatic populations of T cells. We have shown that it is possible to deliver lentiviral shRNA to these CD8 T cells to knock down PD-1 from high to negligible levels of expression. PD-1 knockdown has similarly been achieved by alternative approaches such as CRISPR-Cas9, zinc-finger nucleases, and transcription activator-like effector nucleases (TALENs).[43–46] However, the approach we developed allows genetic editing of targets such as PD-1 to be focused on T cells of desired specificity, making it possible to target low-frequency, endogenously primed responses to tumor and viral antigens. Modifying existing host-derived T cell responses ensures boosting of relevant specificities without the need for cloning personalized T cell receptors. However, in settings in which endogenous responses are too low in frequency, we and others[47,48] demonstrate that it is feasible to instead modify checkpoints such as PD-1 on T cells that have also undergone TCR gene transfer. We selected PD-1 because it is a major regulator of peripheral tolerance and T cell exhaustion in tumors and viral infections that is already being blocked in patients with HCC.[37] Our results provide a paradigm for combining genetic optimization of both specificity and function and/or survival of T cells in the setting of HBV-related HCC, which could be tested on other molecular targets in the future.

PD-1 knockdown boosted some T cell functionality in 2D cultures, but to better assess its impact on adoptive cancer immunotherapy, we implemented a microfluidic model, recreating some characteristics of a solid tumor microenvironment.[28,29,49,50] These types of models have gained attention as disease-relevant *in vitro* platforms, enabling the identification of human cell-cell interactions within a controlled spatio-temporal environment.[28,29,50,51] Because lysis of tumor cells embedded in the 3D collagen microenvironment is a net result of both the chemotactic characteristics and the intrinsic killing capacity of the engineered TCR-redirected T cells, this assay mimics more closely what is encountered physiologically during adoptive T cell therapy of solid tumors, in which these parameters can be a critical determinant of antitumor efficiency. In this case, we specifically recapitulated the high expression of PD-L1 that characterizes the liver and tumor microenvironment by using prototypic proinflammatory cytokines to induce this ligand on both the tumor and the added monocytes. In this setting, we were able to demonstrate the benefit of PD-1 knockdown for boosting the efficiency with which TCR-redirected T cells kill an HBV antigen-expressing tumor. Future studies could test efficacy in animal models, as well as in early human trials of genetically engineered T cells in HCC.[50]

A possible clinical limitation of this directed approach compared to systemic PD-1 antibody blockade is that, paradoxically, non-T cell effects may be of some therapeutic benefit. For example, a study showed that systemic PD-1 blockade can inhibit melanoma cell-intrinsic tumorigenesis, an effect mediated by PD-1-expressing cancer subpopulations and independent of T cell PD-1.[52] Our data demonstrate additional caveats of targeting T cell PD-1, revealing that its knockdown potentiates compensatory increases in other co-inhibitory pathways such as CTLA-4 and Tim-3. Work from the lymphocytic choriomeningitis virus (LCMV) mouse model of chronic viral infection and subsequent patient studies have shown that T cells are co-regulated by multiple layers of inhibitory receptors;[30,53–56] our new data reveal that the expression of individual co-inhibitory receptors can be cross-regulated. This suggests the need to knock down more than one co-inhibitory receptor on T cells, in line with the enhanced responses seen with use of dual checkpoint inhibitors in cancer immunotherapy.[1,3] The heterogeneity and non-redundancy of co-inhibitory receptor hierarchies in patient populations may necessitate personalized approaches.[54–56] Larger studies are needed to test the possibility that the variations in donor-to-donor efficacy of PD-1 blockade we observed in the 3D model can predict responsiveness *in vivo*. An alternative is to combine PD-1 silencing with enhanced co-stimulation, as tested in a murine model of CAR immunotherapy.[15]

In addition to providing insights for the refinement of adoptive cell therapy of HCC, our data underscore the multifaceted role of PD-1 on T cells. We find enhancement of CD8 T cell cytolytic and non-cytolytic antitumor potential following PD-1 knockdown in 2D and 3D cultures, in keeping with its well-accepted role in constraining responses. However, we observe that PD-1 also plays a protective role in the setting of repetitive TCR-dependent stimulation; editing its expression therefore accelerates T cell progression toward apoptosis and end-stage differentiation. This extends to humans the data from the LCMV mouse model, showing a similar pattern of premature cell death and senescence promoted by genetic deletion of T cell PD-1.[32] Although the detailed transcriptional and epigenetic changes underpinning the compensatory alterations we observed remain to be defined, the associated induction of T-bet is in keeping with early changes upon PD-1 knockdown noted in the LCMV system[32] and raises the possibility that lack of PD-1 may accelerate T cell differentiation toward a short-lived effector subtype.[57] Thus, our data add to the accumulating body of evidence that PD-1-driven T cell changes occurring during exhaustion constitute an adaptation that allows T cells to withstand the onslaught of continual antigenic stimulation while maintaining a degree of ongoing control.[31–33] This is supported by our observations that high PD-1 expression is a feature of long-lived, liver-resident T cells[21] and of responses maintaining viral control after treatment interruption in CHB.[34] Altogether, these findings argue that knockdown of PD-1 will be most effective on short-lived T cell populations, because this could boost their antitumor efficacy while avoiding the limitation of premature senescence upon repetitive stimulation. This could be applied in the context of the tested approach for HBV-related HCC of adoptive cell therapy with T cells that have their TCR specificity redirected transiently by mRNA electroporation (ClinicalTrials.gov: NCT02686372 and NCT02719782).[49,50,58]

In summary, we show for the first time the potential to gene-edit T cells specific for viral tumor-related antigens from the periphery and liver of patients with CHB. Although technically challenging, the extraction and transduction of liver-resident T cells could exploit their specific adaptations. However, whether this strategy would promote their long-term survival at the site of hepatic tumors remains to be tested. Our data provide the proof of principle of the capacity to preferentially target and modify antigen-specific T cells using either endogenous or TCR-redirected T cells. PD-1 downregulation increases antitumor cytotoxicity and proliferation but also drives long-term over-activation, resulting in a phenotype with features of apoptosis and accelerated differentiation. Thus, our more focused approach could overcome some problems with systemic PD-1 blockade but uncovers residual limitations related to the complex role of this co-inhibitory receptor in T cells. Our work highlights the need for more fundamental editing of genomic and non-coding mediators of T cell exhaustion to refine adoptive cell therapy for persistent viral infections and cancers. The identification of distinct enhancers differentiating the upregulation of PD-1 in acutely activated T cells, as opposed to chronically exhausted T cells, provides scope for future state-specific modulation.[59] In the interim, the enhanced immediate tumor killing we show in a novel microfluidic 3D-model supports consideration of combining PD-1 knockdown with TCR gene transfer for adoptive cell therapy trials in patients with HCC.

MATERIALS AND METHODS

Subjects and Sample Collection

This study was approved by the local ethical boards of London-Brent (Regional Ethics Committee reference number 16/LO/1699) and Brighton and Sussex (Research Ethics Committee reference number 11/LO/0421) (IRAS Project ID 43993) and the Institutional Review Board of the National University of Singapore. Liver resections and perfusions were obtained through the Tissue Access for Patient Benefit Scheme, approved by the Royal Free Hospital Biobank Ethical Committee (references 11/WA/0077 and 11/H0720/4). A total of thirty-eight chronic HBV patients and nine healthy volunteers participated in the study. All participants were HCV and HIV sero-negative and treatment naive. PBMCs were separated using Ficoll-Paque Plus (GE Healthcare) density gradient centrifugation and cryopreserved. Liver sections from five CHB biopsies, two CHB liver resections, and four healthy margins from colorectal metastasis resections were obtained. Seven transplant perfusates from cadaveric donor livers were collected during liver transplant surgery under the standard graft preparation protocols.

Cell Lines

T2 cells were cultured in RPMI 1640 (Life Technologies) supplemented with 10% heat-inactivated fetal bovine serum (FBS), 0.5 mM sodium pyruvate, 100 U/mL penicillin, 100 μg/mL streptomycin, and GlutaMAX. Phoenix and HEK293T were grown in DMEM (Life Technologies), 10% FBS, 100 U/mL penicillin, 100 μg/mL streptomycin, and GlutaMAX. HepG2preS1 cells expressing the full genotype D envelope gene linked to GFP were obtained as described[28] and maintained in DMEM with 10% heat-inactivated FBS, 20 mM HEPES, 0.5 mM sodium pyruvate, 100 IU/mL penicillin, 100 μg/mL streptomycin, and 5 μg/mL Plasmocin (InvivoGen), with 5 μg/mL puromycin (Clontech) to select for transgene-expressing target cells.

Lentiviral Vector Production

The pHIV1-SIREN lentiviral vector (LV-shRNA) expressing PD-1-specific shRNA (5′-GTGCTAAACTGGTACCGCAT-3′) under a U6 promoter and encoding for EGFP under a phosphoglycerate kinase (PGK) promoter was described previously.[60] LV-shCTR was generated, replacing the shPD-1 sequence for a control shRNA (5′-CCTAAGGTTAAGTCGCCCTCG-3′), and mCherry constructs were generated by GenScript (USA). Three-plasmid co-transfection into HEK293T cells was used to make VSV-g pseudotyped lentivirus. The 12e6 HEK293T cells were seeded in 150-mm plates; 24 hr later, they were transfected using Fugene6 (Promega) with the following the plasmids: 3.75 μg SIN pHV (vector plasmid), 2.5 μg p8.91 (Gag-Pol expression plasmid), and 2.5 μg pMD.G (VSV-g env expression plasmid). Supernatant was collected over 24-hr periods for 3 days. Following collection, LV supernatant was passed through a 0.45-μm filter and concentrated by ultracentrifugation in a SureSpin 630 rotor (Thermo Scientific, Waltham, MA) through a 25% sucrose cushion at 25.000 rpm for 2 hr at 4°C. The pellet was resuspended in PBS and stored at −80°C. Lentivirus vectors were added at a MOI of 20. Lentivectors were titrated as described.[60]

Lentiviral Transduction Protocol for Antigen-Specific CD8 T Cells

PBMCs from HLA-A2+ healthy donors and CHB patients were isolated by Ficoll-Hypaque gradient separation (Lymphoprep) and enriched for CD8+ cells with the human CD8 MicroBead Isolation Kit (Miltenyi Biotec) following the manufacturer's instructions. CD8 T cells were stimulated with the following peptides for 24 hr in RPMI-10% FBS supplemented with IL-2 (20 U/mL) in the presence or absence of IL-15 (10 ng/mL, R&D): 1 μM HBV-derived HLA-A2-restricted epitopes (core FLPSDFFPSV; envelope FLLTRILTI, WLSLLVPFV, LLVPFVQWFV, and GLSPTVWLSV; and polymerase GLSRYVARL and KLHLYSHPI) (Proimmune) and 1 μM NLVPMVATV (Proimmune). Lentiviral vectors were added 24 hr after stimulation, and media were replaced with RPMI-10% FBS plus IL-2 (20 U/mL) 72 hr later. Functional studies were carried out 5 days post-transduction. T2 cells were loaded with 1 μg/mL HBV or CMV peptides for 1 hr at 37°C, washed, and co-cultured with lentivirally modified T cells (ratio 1:1) overnight in RPMI-10% FBS with 1 μg/mL brefeldin A (BFA) (Sigma). GFP and mCherry expression were used as markers for lentiviral transduction. Virus-specific responses were identified by IFN-γ production. Whole PBMCs from patients with CHB were stimulated for 24 hr, with 1 μM overlapping peptides (OLPs) spanning the whole HBV core protein, genotype D (AYW) (JPT Peptide Technologies) in the presence of IL-15 (10 ng/mL). On day 9, PBMCs were restimulated with 1 μM OLPs in the presence of BFA.

Retroviral Transduction

The antigen-specific encoding for HBc_{18-27}-specific TCR Va3/Vb8.2 chains (c18-TCR), the HBs_{183-91}-specific TCR (s183-TCR) Va34/Vb28, and the amphotropic envelope plasmid (pCMV-Ampho) were described previously.[14,25] 15e6 Phoenix amphotropic packaging cells were seeded into 150-mm tissue culture dishes 24 hr before transfection. Phoenix cells were transiently co-transfected using Fugene6 (Promega) with 3.75 μg of MP71-TCR, together with 2.5 μg of pCMV-Ampho. 24 hr later, DMEM was replaced with fresh medium and retroviral supernatants were collected and spun down 72 hr after transfection. PBMC were stimulated with 600 U/mL IL-2 (PeproTech), 0.5 μg/mL plate-bound α-CD3 (OKT-3; eBioscience), and 1 μg/mL α-CD28 for 48 hr. Untreated 24-well tissue culture plates were coated with 30 μg/mL RetroNectin (Takara Bio) overnight at 4°C one day before transduction. Wells were then washed with PBS and blocked with PBS 2% BSA. 1.5–2 mL of retroviral supernatant was added per well and spun down at 2,000 × *g* for 2 hr at 37°C. Lymphocytes were harvested, washed, and counted, and 1e6 cells were plated into RetroNectin-coated wells with IL-2 (600 U/mL) and mixed with the retroviral supernatant. After 24 hr, medium was replaced and cells were maintained in RPMI-10% FBS plus 100 U/mL IL-2. TCR surface expression was tested with HLA-A201-HBc_{18-27}-PE and HLA-A201-HBe_{183-91}-PE dextramers (Proimmune) or anti-Cβ antibodies 3–5 days after transduction. Functionality of TCR-redirected T cells was tested 7 days after transduction, as described later. Flow cytometry was performed using a FACS LSRII and Fortessa flow cytometer (BD Biosciences), and data were analyzed with the FACS Diva program (BD Biosciences). In some cases, HBV TCR retrovirus supernatant was concentrated and titrated as described for lentiviral particles and frozen at −80°C.

RNA Extraction and Real-Time qPCR

PBMCs from healthy donors were lentivirally transduced with LV-shCTR-mCherry and LV-shPD-1-mCherry following the previously described protocol. mCherry+ CD8+ and CD4+ T cells were sorted in a BD FACS Aria, and cell pellets used for RNA extraction with the RNeasy micro-RNA Purification Kit (QIAGEN). Retrotranscription was performed with the iScript cDNA Synthesis kit (Bio-Rad). PD-1 expression was quantified using IQ SYBR Green Supermix (Bio-Rad) in the Roche LightCycler-480 qPCR machine, with B-actin expression for normalization.

Intrahepatic Lymphocyte Isolation and Lentiviral Transduction

Liver tissue was digested using enzymes (1 ng/mL collagenase IV and 0.1 ng/mL DNase I) and mechanical maceration before passing through a 70-μm cell strainer. Intrahepatic leukocytes isolated from liver biopsies were used immediately, whereas those isolated from larger liver tissue and perfusates were isolated with an extra Percoll density centrifugation step before experimentation. T cells from non-HBV livers were activated for 24 hr with IL-15 and IL-7 (10 ng/mL) before the addition of LV particles, whereas T cells from CHB samples were activated with 1 μM HBV core OLPs or HLA-A2-restricted HBV peptides for HLA-A2+ CHB patients plus IL-15 and IL-7 (10 ng/mL) and IL-2 (20 IU/mL). 72 hr after activation, media were replaced by maintaining media (MM) containing RPMI-10% FBS with IL-2 (100 U/mL) and IL-15 and IL-7 (5 ng/mL).

Generation of shPD-1/HBV TCR CD8 T Cells

TCR-redirected CD8 T cells from healthy controls were sorted based on TCR Cβ expression in FACS Aria (BD Biosciences) and expanded with IL-2 (100 U/mL) and IL-15 and IL-7 (5 ng/mL). After 1–2 weeks, CD8 T cells were lentivirally transduced after 24 hr of stimulation with 0.5 μg/mL plate-bound α-CD3 (OKT-3; eBioscience) and 1 μg/mL α-CD28 plus IL-2 (600 U/mL) and IL-15 and IL-7 (10 ng/mL). Three days after activation, media were replaced with MM, and functional analysis was performed 5–7 days after lentiviral transduction.

Function of TCR-Transduced T Cells

Redirected T cell functionality was tested 7 days after transduction. HLA-A0201 T2 cells were loaded with 1 μg/mL of each peptide for 1 hr at 37°C, washed, and co-cultured with TCR-redirected T cells overnight in RPMI-10% FBS with 1 μg/mL BFA (Sigma) in a 1:1 ratio.

Production of s183–191 TCR mRNA and Electroporation Procedures

HBV-$env_{183-191}$TCR mRNA was obtained as described.[49] HBV-env_{183-91}-specific TCR DNA was subcloned into the pVAX1 vector, linearized using FastDigest XbaI (Thermo Fisher Scientific), and used to produce the TCR-mRNA using the Ambion mMESSAGE mMACHINE T7 Ultra kit (Thermo Fisher Scientific). For electroporation with the nucleofector device (BTX, Agilpulse Max), $5e10^6$–10e106 peripheral blood mononuclear cells were activated for 24 hr with 0.5 μg/mL plate-bound α-CD3 (OKT-3; eBioscience) and 1 μg/mL α-CD28 plus IL-2 (600 U/mL) and IL-15 and IL-7 (10 ng/mL) in AIM-V 2% human AB serum. Lentiviral vectors were added 24 hr after stimulation with a MOI of 10, and media were replaced on day 3 with AIM-V 2% human AB serum plus 100 U/mL IL-2. PBMCs were activated on day 7 under the same conditions. On day 13, PBMCs were placed overnight with AIM-V 2% human AB serum plus 1,000 U/mL IL-2. On day 14, PBMCs were resuspended in 100–200 μL of Cell Line Nucleofector Solution V (Lonza) and TCR mRNA was added at 100 μg/mL. The mixture was placed in a certified cuvette (BTX) and electroporated. After electroporation, cells were resuspended in AIM-V 2% human AB serum plus 100 IU/mL recombinant IL-2 (rIL-2).

Staining for Multimer+ T Cells and Intracellular Cytokine Production

HBV-specific T cells were identified with a pool of HLA-A2/peptide dextramers: core FLPSDFFPSV; envelope FLLTRILTI, WLSLLVPFV, and GLSPTVWLSV; and polymerase GLSRYVARL and KLHLYSHPI. CMV-specific dextramers were loaded with NLVPMVATV. Cells were stained with dextramers, washed, and rested for 30 min before proceeding to flow cytometry analysis or were stimulated with peptide-loaded T2 cells for 1 hr before the addition of BFA in a 1:1 ratio. After 5 hr of incubation, cells were stained with the surface marker antibodies, live-dead staining and anti-CD19

(BD Biosciences), to exclude dead cells and non-specific binding, respectively. Intracellular cytokines were detected as described.

Functional Studies

PBMCs from CHB patients were stimulated for 24 hr with 0.5 μg/mL plate-bound α-CD3 and 1 μg/mL α-CD28 (eBioscience) before the addition of lentiviral particles. Three days after activation, media were changed with fresh media containing IL-15 and IL-7 (5 ng/mL). Seven days after lentiviral transduction, T cells were expanded with α-CD3/CD28 antibodies. Fifteen days after lentiviral transduction, overnight restimulation was carried out in the presence of 1 μg/mL BFA. All cultures were carried out in the presence of 20 IU/mL recombinant human IL-2 (Miltenyi Biotec) in RPMI-10% FBS at 37°C.

Flow Cytometric Analysis

9- or 12-color flow cytometry was used for all experiments. Dead cells were always excluded using a live-dead fixable dye staining kit (Invitrogen). Cells were stained for surface markers CD3, CD4, CD8 (eBioscience), PD-1 (BioLegend), 2B4, Tim3, CD57, VBm, PD-L1, and CD14. Cells were then fixed and permeabilized, and intracellular molecules were detected using anti-IFN-γ, TNF-α, granzyme B, and CTLA-4. For annexin V quantification, following surface marker staining and live-dead staining, cells were washed and annexin V staining was performed in annexin V binding buffer (BioLegend). For Ki67 intracellular or nuclear staining, fixation was performed with CytoFix buffer (BD Biosciences) and permeabilization using 0.01% Triton X-100 solution. All samples were acquired on a BD LSRII or BD Fortessa. All analysis was performed using FlowJo (Tree Star).

3D Microdevice Assay

The embedding of HepG2-preS1 targets and monocytes in microdevices was described previously.[28,29] A commercially available microfluidic device (DAX-1, AIM Biotech) was used to co-culture genetically modified T cells with HepG2-preS1 and in the presence or absence of CD14+ cells. Two collagen hydrogels were prepared for injection in the dedicated microfluidic region: (1) 2.5 mg/mL type I collagen gel solution containing 20 μL homogenously dissociated freshly trypsinized HepGpreS1 at 50e6 cells/mL, mixed with 20 μL 10× PBS, 4 μL NaOH (0.5 N), 129.2 μL collagen type I (Corning), and 22.9 μL cell culture water, and (2) the preceding hydrogel with the addition of CD14+ cells (isolated from fresh PBMCs with a CD14 microbead kit according to manufacturer's instructions), and 20 μL of a 50e6 cells/mL labeled cell solution (orange CMRA, Thermo Fisher Scientific). 10 μL hydrogel master mix was used for injection in the dedicated device region, maintained in the holder at 37°C for 40 min to allow for gel polymerization by thermal cross-linking. After gel polymerization, media channels were filled with complete RPMI in the presence or absence of 1,000 U/mL IFN-γ, 100 ng/mL TNF-α, and 100 U/mL IL-2. DRAQ7 (BioLegend) was added to the media at a concentration of 3 μM to discriminate between live and dead cells. Devices were incubated for 24 hr to permit interaction of HepG2preS1 target cells with collagen matrix and set the microenvironment. TCR-redirected T cells (shPD-1 or shCTR), labeled with 3 μM CellTracker Violet BMQC (Life Technologies, Carlsbad, CA) resuspended in RPMI-10% FBS at 6e6 cells/mL, were added (30 μL) into one of the two media channels flanking the central gel region of each device and incubated for 15 hr at 37°C before image acquisition.

3D Device Imaging and Data Analysis

Confocal images of constant hydrogel regions were taken on day 1 before the addition of T cells and 24 hr after T cell injection in the device. The images were acquired with either LSM7800 confocal microscopy (Zeiss) or high-content imaging-system (Operetta, PerkinElmer) and analyzed using Imaris software (Bitplane) to quantify dead targets before and after T cell addition. Live targets were recognized by GFP expression in green, dead cells were identified by DRAQ7 in red, T cells were labeled with BMQC in blue, and CD14+ cells were labeled with orange CMRA stain. The number of killed targets was calculated by subtraction of baseline dead cells before T cell addition and normalized to allow comparison between donors with variable amounts of HBV TCR expression, such that the baseline control condition was set to 100%.

Extraction of Cells from the 3D Device

Media from the 3D device ports were replaced by a solution of 1 mg/mL collagenase type II (Gibco) and incubated for 5 min at 37°C, and then cell suspensions were extracted from the gel and media channels. The 3D device was washed once with PBS to flush out remaining cells. Cells were then washed with PBS and stained for flow cytometry.

Statistical Analysis

Statistical analyses were performed using one-way ANOVA, Student's t test and Tukey's test, the non-parametric Wilcoxon matched pairs test, Friedman test and Dunn's multiple comparison tests, and Pearson product-moment correlation coefficient as appropriate. Significant differences were marked in figures legends: †$p < 0.1$, *$p < 0.05$, **$p < 0.01$, and ***$p < 0.001$. Any p value less than 0.001 was designated with three asterisks.

SUPPLEMENTAL INFORMATION

Supplemental Information includes four figures and can be found with this article online at https://doi.org/10.1016/j.ymthe.2018.08.013.

AUTHOR CONTRIBUTIONS

I.O. and M.K.M. conceived the project; I.O., A.S., F.A.V., A.B., A.P., and M.K.M. designed the experiments; I.O., A.P., and H.S. generated data; I.O., A.P., and M.K.M. analyzed data; F.A.V., S.-A.X., H.S., A.B., and D.E. provided essential molecular tools; F.R., N.H., G.F., B.R.D., P.T.F.K., and M.K.M. provided essential patient samples; I.O. and M.K.M. prepared the manuscript; and all other authors provided critical review of the manuscript.

CONFLICTS OF INTEREST

M.K.M. collaborates and receives research support from Gilead Sciences, F. Hoffmann-La Roche, and Immunocore and serves as a

consultant or on advisory boards for F. Hoffmann-La Roche, Gilead Sciences, Immunocore, Arbutus Biopharma, and Jannsen. A.B. collaborates and receives research support from Gilead Sciences; serves as a consultant or on advisory boards for Gilead Sciences, MedImmune, IONIS, Abivax, and HUMABS BioMed; and is a co-founder of LION TCR. A.P. is a consultant for AIM Biotech.

ACKNOWLEDGMENTS

This work was funded by a Wellcome Senior Investigator Award (101849/Z/13/Z) and Medical Research Council UK grant (G0801213) to M.K.M. I.O. was funded by an EASL post-doctoral fellowship, D.E. is funded by a Miguel Servet Fellowship (CP12/03114, Insituto de Salud Carlos III, Spain), H.S. is funded by an LRF Programme grant, and A.P. is funded by a National Research Foundation (NRF) grant (NRF-CRP17-2017-06). We are grateful to all patients and control volunteers who participated in this study and to all clinical staff who helped with participant recruitment, including Amir Gander and the Tissue Access for Patient Benefit project at The Royal Free Hospital.

REFERENCES

1. Wherry, E.J., and Kurachi, M. (2015). Molecular and cellular insights into T cell exhaustion. Nat. Rev. Immunol. *15*, 486–499.
2. Boussiotis, V.A. (2016). Molecular and biochemical aspects of the PD-1 checkpoint pathway. N. Engl. J. Med. *375*, 1767–1778.
3. Sharma, P., and Allison, J.P. (2015). The future of immune checkpoint therapy. Science *348*, 56–61.
4. Gubin, M.M., Zhang, X., Schuster, H., Caron, E., Ward, J.P., Noguchi, T., Ivanova, Y., Hundal, J., Arthur, C.D., Krebber, W.J., et al. (2014). Checkpoint blockade cancer immunotherapy targets tumour-specific mutant antigens. Nature *515*, 577–581.
5. Rizvi, N.A., Hellmann, M.D., Snyder, A., Kvistborg, P., Makarov, V., Havel, J.J., Lee, W., Yuan, J., Wong, P., Ho, T.S., et al. (2015). Cancer immunology. Mutational landscape determines sensitivity to PD-1 blockade in non-small cell lung cancer. Science *348*, 124–128.
6. Tumeh, P.C., Harview, C.L., Yearley, J.H., Shintaku, I.P., Taylor, E.J., Robert, L., Chmielowski, B., Spasic, M., Henry, G., Ciobanu, V., et al. (2014). PD-1 blockade induces responses by inhibiting adaptive immune resistance. Nature *515*, 568–571.
7. Naidoo, J., Page, D.B., Li, B.T., Connell, L.C., Schindler, K., Lacouture, M.E., Postow, M.A., and Wolchok, J.D. (2015). Toxicities of the anti-PD-1 and anti-PD-L1 immune checkpoint antibodies. Ann. Oncol. *26*, 2375–2391.
8. Franceschini, D., Paroli, M., Francavilla, V., Videtta, M., Morrone, S., Labbadia, G., Cerino, A., Mondelli, M.U., and Barnaba, V. (2009). PD-L1 negatively regulates CD4+CD25+Foxp3+ Tregs by limiting STAT-5 phosphorylation in patients chronically infected with HCV. J. Clin. Invest. *119*, 551–564.
9. Bertoletti, A., Brunetto, M., Maini, M.K., Bonino, F., Qasim, W., and Stauss, H. (2015). T cell receptor-therapy in HBV-related hepatocellularcarcinoma. OncoImmunology *4*, e1008354.
10. Boni, C., Fisicaro, P., Valdatta, C., Amadei, B., Di Vincenzo, P., Giuberti, T., Laccabue, D., Zerbini, A., Cavalli, A., Missale, G., et al. (2007). Characterization of hepatitis B virus (HBV)-specific T-cell dysfunction in chronic HBV infection. J. Virol. *81*, 4215–4225.
11. Fisicaro, P., Valdatta, C., Massari, M., Loggi, E., Biasini, E., Sacchelli, L., Cavallo, M.C., Silini, E.M., Andreone, P., Missale, G., and Ferrari, C. (2010). Antiviral intrahepatic T-cell responses can be restored by blocking programmed death-1 pathway in chronic hepatitis B. Gastroenterology *138*, 682–693.
12. Gehring, A.J., Ho, Z.Z., Tan, A.T., Aung, M.O., Lee, K.H., Tan, K.C., Lim, S.G., and Bertoletti, A. (2009). Profile of tumor antigen-specific CD8 T cells in patients with hepatitis B virus-related hepatocellular carcinoma. Gastroenterology *137*, 682–690.
13. Schmidt, N., and Thimme, R. (2016). Role of immunity in pathogenesis and treatment of hepatocellular carcinoma. Dig. Dis. *34*, 429–437.
14. Qasim, W., Brunetto, M., Gehring, A.J., Xue, S.A., Schurich, A., Khakpoor, A., Zhan, H., Ciccorossi, P., Gilmour, K., Cavallone, D., et al. (2015). Immunotherapy of HCC metastases with autologous T cell receptor redirected T cells, targeting HBsAg in a liver transplant patient. J. Hepatol. *62*, 486–491.
15. Cherkassky, L., Morello, A., Villena-Vargas, J., Feng, Y., Dimitrov, D.S., Jones, D.R., Sadelain, M., and Adusumilli, P.S. (2016). Human CAR T cells with cell-intrinsic PD-1 checkpoint blockade resist tumor-mediated inhibition. J. Clin. Invest. *126*, 3130–3144.
16. John, L.B., Devaud, C., Duong, C.P., Yong, C.S., Beavis, P.A., Haynes, N.M., Chow, M.T., Smyth, M.J., Kershaw, M.H., and Darcy, P.K. (2013). Anti-PD-1 antibody therapy potently enhances the eradication of established tumors by gene-modified T cells. Clin. Cancer Res. *19*, 5636–5646.
17. Abate-Daga, D., Hanada, K., Davis, J.L., Yang, J.C., Rosenberg, S.A., and Morgan, R.A. (2013). Expression profiling of TCR-engineered T cells demonstrates overexpression of multiple inhibitory receptors in persisting lymphocytes. Blood *122*, 1399–1410.
18. Gargett, T., Yu, W., Dotti, G., Yvon, E.S., Christo, S.N., Hayball, J.D., Lewis, I.D., Brenner, M.K., and Brown, M.P. (2016). GD2-specific CAR T cells undergo potent activation and deletion following antigen encounter but can be protected from activation-induced cell death by PD-1 blockade. Mol. Ther. *24*, 1135–1149.
19. Dong, H., Strome, S.E., Salomao, D.R., Tamura, H., Hirano, F., Flies, D.B., Roche, P.C., Lu, J., Zhu, G., Tamada, K., et al. (2002). Tumor-associated B7-H1 promotes T-cell apoptosis: a potential mechanism of immune evasion. Nat. Med. *8*, 793–800.
20. Kassel, R., Cruise, M.W., Iezzoni, J.C., Taylor, N.A., Pruett, T.L., and Hahn, Y.S. (2009). Chronically inflamed livers up-regulate expression of inhibitory B7 family members. Hepatology *50*, 1625–1637.
21. Pallett, L.J., Davies, J., Colbeck, E.J., Robertson, F., Hansi, N., Easom, N.J.W., Burton, A.R., Stegmann, K.A., Schurich, A., Swadling, L., et al. (2017). IL-2^{high} tissue-resident T cells in the human liver: Sentinels for hepatotropic infection. J. Exp. Med. *214*, 1567–1580.
22. Ganesan, A.P., Clarke, J., Wood, O., Garrido-Martin, E.M., Chee, S.J., Mellows, T., Samaniego-Castruita, D., Singh, D., Seumois, G., Alzetani, A., et al. (2017). Tissue-resident memory features are linked to the magnitude of cytotoxic T cell responses in human lung cancer. Nat. Immunol. *18*, 940–950.
23. Korin, Y.D., and Zack, J.A. (1998). Progression to the G1b phase of the cell cycle is required for completion of human immunodeficiency virus type 1 reverse transcription in T cells. J. Virol. *72*, 3161–3168.
24. Di Scala, M., Otano, I., Gil-Fariña, I., Vanrell, L., Hommel, M., Olagüe, C., Vales, A., Galarraga, M., Guembe, L., Ortiz de Solorzano, C., et al. (2016). Complementary effects of interleukin-15 and alpha interferon induce immunity in hepatitis B virus transgenic mice. J. Virol. *90*, 8563–8574.
25. Gehring, A.J., Xue, S.A., Ho, Z.Z., Teoh, D., Ruedl, C., Chia, A., Koh, S., Lim, S.G., Maini, M.K., Stauss, H., and Bertoletti, A. (2011). Engineering virus-specific T cells that target HBV infected hepatocytes and hepatocellular carcinoma cell lines. J. Hepatol. *55*, 103–110.
26. Dudley, M.E., Wunderlich, J.R., Shelton, T.E., Even, J., and Rosenberg, S.A. (2003). Generation of tumor-infiltrating lymphocyte cultures for use in adoptive transfer therapy for melanoma patients. J. Immunother. *26*, 332–342.
27. Bennett, F., Luxenberg, D., Ling, V., Wang, I.M., Marquette, K., Lowe, D., Khan, N., Veldman, G., Jacobs, K.A., Valge-Archer, V.E., et al. (2003). Program death-1 engagement upon TCR activation has distinct effects on costimulation and cytokine-driven proliferation: attenuation of ICOS, IL-4, and IL-21, but not CD28, IL-7, and IL-15 responses. J. Immunol. *170*, 711–718.
28. Pavesi, A., Tan, A.T., Koh, S., Chia, A., Colombo, M., Antonecchia, E., Miccolis, C., Ceccarello, E., Adriani, G., Raimondi, M.T., et al. (2017). A 3D microfluidic model for preclinical evaluation of TCR-engineered T cells against solid tumors. JCI Insight *2*, e89762.
29. Lee, S.W.L., Adriani, G., Ceccarello, E., Pavesi, A., Tan, A.T., Bertoletti, A., Kamm, R.D., and Wong, S.C. (2018). Characterizing the role of monocytes in T cell cancer immunotherapy using a 3D microfluidic model. Front. Immunol. *9*, 416.

30. Blackburn, S.D., Shin, H., Haining, W.N., Zou, T., Workman, C.J., Polley, A., Betts, M.R., Freeman, G.J., Vignali, D.A., and Wherry, E.J. (2009). Coregulation of CD8+ T cell exhaustion by multiple inhibitory receptors during chronic viral infection. Nat. Immunol. *10*, 29–37.

31. Utzschneider, D.T., Legat, A., Fuertes Marraco, S.A., Carrié, L., Luescher, I., Speiser, D.E., and Zehn, D. (2013). T cells maintain an exhausted phenotype after antigen withdrawal and population reexpansion. Nat. Immunol. *14*, 603–610.

32. Odorizzi, P.M., Pauken, K.E., Paley, M.A., Sharpe, A., and Wherry, E.J. (2015). Genetic absence of PD-1 promotes accumulation of terminally differentiated exhausted CD8+ T cells. J. Exp. Med. *212*, 1125–1137.

33. Zehn, D., Utzschneider, D.T., and Thimme, R. (2016). Immune-surveillance through exhausted effector T-cells. Curr. Opin. Virol. *16*, 49–54.

34. Rivino, L., Le Bert, N., Gill, U.S., Kunasegaran, K., Cheng, Y., Tan, D.Z., Becht, E., Hansi, N.K., Foster, G.R., Su, T.H., et al. (2018). Hepatitis B virus-specific T cells associate with viral control upon nucleos(t)ide-analogue therapy discontinuation. J. Clin. Invest. *128*, 668–681.

35. Isogawa, M., Furuichi, Y., and Chisari, F.V. (2005). Oscillating CD8(+) T cell effector functions after antigen recognition in the liver. Immunity *23*, 53–63.

36. Iwai, Y., Terawaki, S., Ikegawa, M., Okazaki, T., and Honjo, T. (2003). PD-1 inhibits antiviral immunity at the effector phase in the liver. J. Exp. Med. *198*, 39–50.

37. El-Khoueiry, A.B., Sangro, B., Yau, T., Crocenzi, T.S., Kudo, M., Hsu, C., Kim, T.Y., Choo, S.P., Trojan, J., Welling, T.H., et al. (2017). Nivolumab in patients with advanced hepatocellular carcinoma (CheckMate 040): an open-label, non-comparative, phase 1/2 dose escalation and expansion trial. Lancet *389*, 2492–2502.

38. Gane, E., Gaggar, A., Nguyen, A.H., Subramanian, G.M., McHutchison, J.G., Schwabe, C., and Dunbar, R. (2017). A phase1 study evaluating anti-PD-1 treatment with or without GS-4774 in HBeAg negative chronic hepatitis B patients. J. Hepatol. *66*, S26–S27.

39. Gardiner, D., Lalezari, J., Lawitz, E., DiMicco, M., Ghalib, R., Reddy, K.R., Chang, K.M., Sulkowski, M., Marro, S.O., Anderson, J., et al. (2013). A randomized, double-blind, placebo-controlled assessment of BMS-936558, a fully human monoclonal antibody to programmed death-1 (PD-1), in patients with chronic hepatitis C virus infection. PLoS ONE *8*, e63818.

40. Nishimura, H., Nose, M., Hiai, H., Minato, N., and Honjo, T. (1999). Development of lupus-like autoimmune diseases by disruption of the PD-1 gene encoding an ITIM motif-carrying immunoreceptor. Immunity *11*, 141–151.

41. Topalian, S.L., Hodi, F.S., Brahmer, J.R., Gettinger, S.N., Smith, D.C., McDermott, D.F., Powderly, J.D., Carvajal, R.D., Sosman, J.A., Atkins, M.B., et al. (2012). Safety, activity, and immune correlates of anti-PD-1 antibody in cancer. N. Engl. J. Med. *366*, 2443–2454.

42. Herbst, R.S., Soria, J.C., Kowanetz, M., Fine, G.D., Hamid, O., Gordon, M.S., Sosman, J.A., McDermott, D.F., Powderly, J.D., Gettinger, S.N., et al. (2014). Predictive correlates of response to the anti-PD-L1 antibody MPDL3280A in cancer patients. Nature *515*, 563–567.

43. Beane, J.D., Lee, G., Zheng, Z., Mendel, M., Abate-Daga, D., Bharathan, M., Black, M., Gandhi, N., Yu, Z., Chandran, S., et al. (2015). Clinical scale zinc finger nuclease-mediated gene editing of PD-1 in tumor infiltrating lymphocytes for the treatment of metastatic melanoma. Mol. Ther. *23*, 1380–1390.

44. Menger, L., Sledzinska, A., Bergerhoff, K., Vargas, F.A., Smith, J., Poirot, L., Pule, M., Hererro, J., Peggs, K.S., and Quezada, S.A. (2016). TALEN-mediated inactivation of PD-1 in tumor-reactive lymphocytes promotes intratumoral T-cell persistence and rejection of established tumors. Cancer Res. *76*, 2087–2093.

45. Schumann, K., Lin, S., Boyer, E., Simeonov, D.R., Subramaniam, M., Gate, R.E., Haliburton, G.E., Ye, C.J., Bluestone, J.A., Doudna, J.A., and Marson, A. (2015). Generation of knock-in primary human T cells using Cas9 ribonucleoproteins. Proc. Natl. Acad. Sci. USA *112*, 10437–10442.

46. Su, S., Hu, B., Shao, J., Shen, B., Du, J., Du, Y., Zhou, J., Yu, L., Zhang, L., Chen, F., et al. (2016). CRISPR-Cas9 mediated efficient PD-1 disruption on human primary T cells from cancer patients. Sci. Rep. *6*, 20070.

47. Ren, J., Liu, X., Fang, C., Jiang, S., June, C.H., and Zhao, Y. (2017). Multiplex genome editing to generate universal CAR T cells resistant to PD1 inhibition. Clin. Cancer Res. *23*, 2255–2266.

48. Georgiadis, C., Preece, R., Nickolay, L., Etuk, A., Petrova, A., Ladon, D., Danyi, A., Humphryes-Kirilov, N., Ajetunmobi, A., Kim, D., et al. (2018). Long terminal repeat CRISPR-CAR-coupled "universal" T cells mediate potent anti-leukemic effects. Mol. Ther. *26*, 1215–1227.

49. Koh, S., Shimasaki, N., and Bertoletti, A. (2016). Redirecting T cell specificity using T cell receptor messenger RNA electroporation. Methods Mol. Biol. *1428*, 285–296.

50. Koh, S., Kah, J., Tham, C.Y.L., Yang, N., Ceccarello, E., Chia, A., Chen, M., Khakpoor, A., Pavesi, A., Tan, A.T., et al. (2018). Nonlytic lymphocytes engineered to express virus-specific T-cell receptors limit HBV infection by activating APOBEC3. Gastroenterology *155*, 180–193.

51. Adriani, G., Pavesi, A., Tan, A.T., Bertoletti, A., Thiery, J.P., and Kamm, R.D. (2016). Microfluidic models for adoptive cell-mediated cancer immunotherapies. Drug Discov. Today *21*, 1472–1478.

52. Kleffel, S., Posch, C., Barthel, S.R., Mueller, H., Schlapbach, C., Guenova, E., Elco, C.P., Lee, N., Juneja, V.R., Zhan, Q., et al. (2015). Melanoma cell-intrinsic PD-1 receptor functions promote tumor growth. Cell *162*, 1242–1256.

53. Anderson, A.C., Joller, N., and Kuchroo, V.K. (2016). Lag-3, Tim-3, and TIGIT: co-inhibitory receptors with specialized functions in immune regulation. Immunity *44*, 989–1004.

54. Nebbia, G., Peppa, D., Schurich, A., Khanna, P., Singh, H.D., Cheng, Y., Rosenberg, W., Dusheiko, G., Gilson, R., ChinAleong, J., et al. (2012). Upregulation of the Tim-3/galectin-9 pathway of T cell exhaustion in chronic hepatitis B virus infection. PLoS ONE *7*, e47648.

55. Schurich, A., Khanna, P., Lopes, A.R., Han, K.J., Peppa, D., Micco, L., Nebbia, G., Kennedy, P.T., Geretti, A.M., Dusheiko, G., et al. (2011). Role of the coinhibitory receptor cytotoxic T lymphocyte antigen-4 on apoptosis-prone CD8 T cells in persistent hepatitis B virus infection. Hepatology *53*, 1494–1503.

56. Bengsch, B., Martin, B., and Thimme, R. (2014). Restoration of HBV-specific CD8+ T cell function by PD-1 blockade in inactive carrier patients is linked to T cell differentiation. J. Hepatol. *61*, 1212–1219.

57. Joshi, N.S., Cui, W., Chandele, A., Lee, H.K., Urso, D.R., Hagman, J., Gapin, L., and Kaech, S.M. (2007). Inflammation directs memory precursor and short-lived effector CD8(+) T cell fates via the graded expression of T-bet transcription factor. Immunity *27*, 281–295.

58. Kah, J., Koh, S., Volz, T., Ceccarello, E., Allweiss, L., Lütgehetmann, M., Bertoletti, A., and Dandri, M. (2017). Lymphocytes transiently expressing virus-specific T cell receptors reduce hepatitis B virus infection. J. Clin. Invest. *127*, 3177–3188.

59. Sen, D.R., Kaminski, J., Barnitz, R.A., Kurachi, M., Gerdemann, U., Yates, K.B., Tsao, H.W., Godec, J., LaFleur, M.W., Brown, F.D., et al. (2016). The epigenetic landscape of T cell exhaustion. Science *354*, 1165–1169.

60. Escors, D., Lopes, L., Lin, R., Hiscott, J., Akira, S., Davis, R.J., and Collins, M.K. (2008). Targeting dendritic cell signaling to regulate the response to immunization. Blood *111*, 3050–3061.

YMTHE, Volume *26*

Supplemental Information

Molecular Recalibration of PD-1+

Antigen-Specific T Cells from Blood and Liver

Itziar Otano, David Escors, Anna Schurich, Harsimran Singh, Francis Robertson, Brian R. Davidson, Giuseppe Fusai, Frederick A. Vargas, Zhi M.D. Tan, Jia Y.J. Aw, Navjyot Hansi, Patrick T.F. Kennedy, Shao-An Xue, Hans J. Stauss, Antonio Bertoletti, Andrea Pavesi, and Mala K. Maini

Supplementary Figure 1

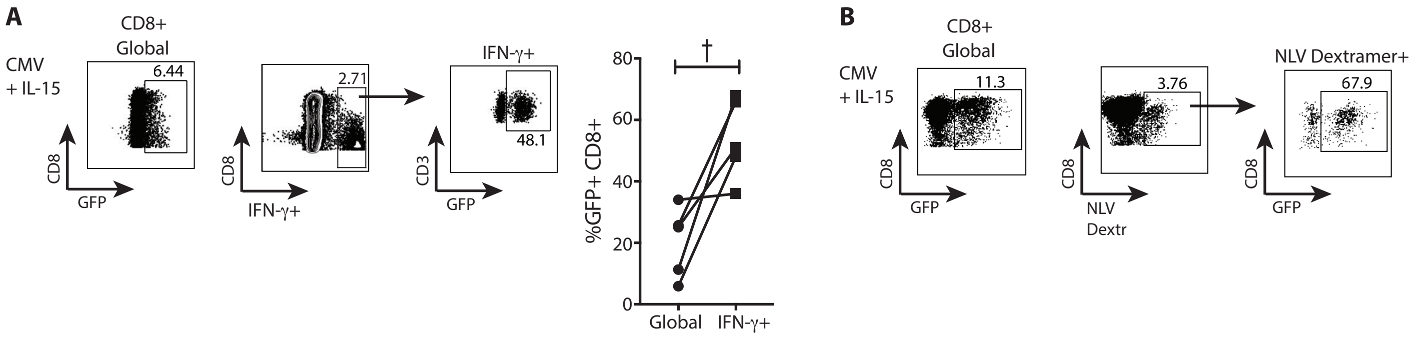

Supplementary Figure 2

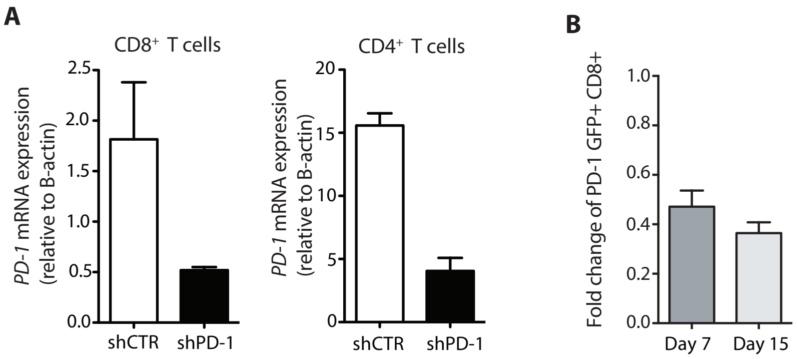

Supplementary Figure 3

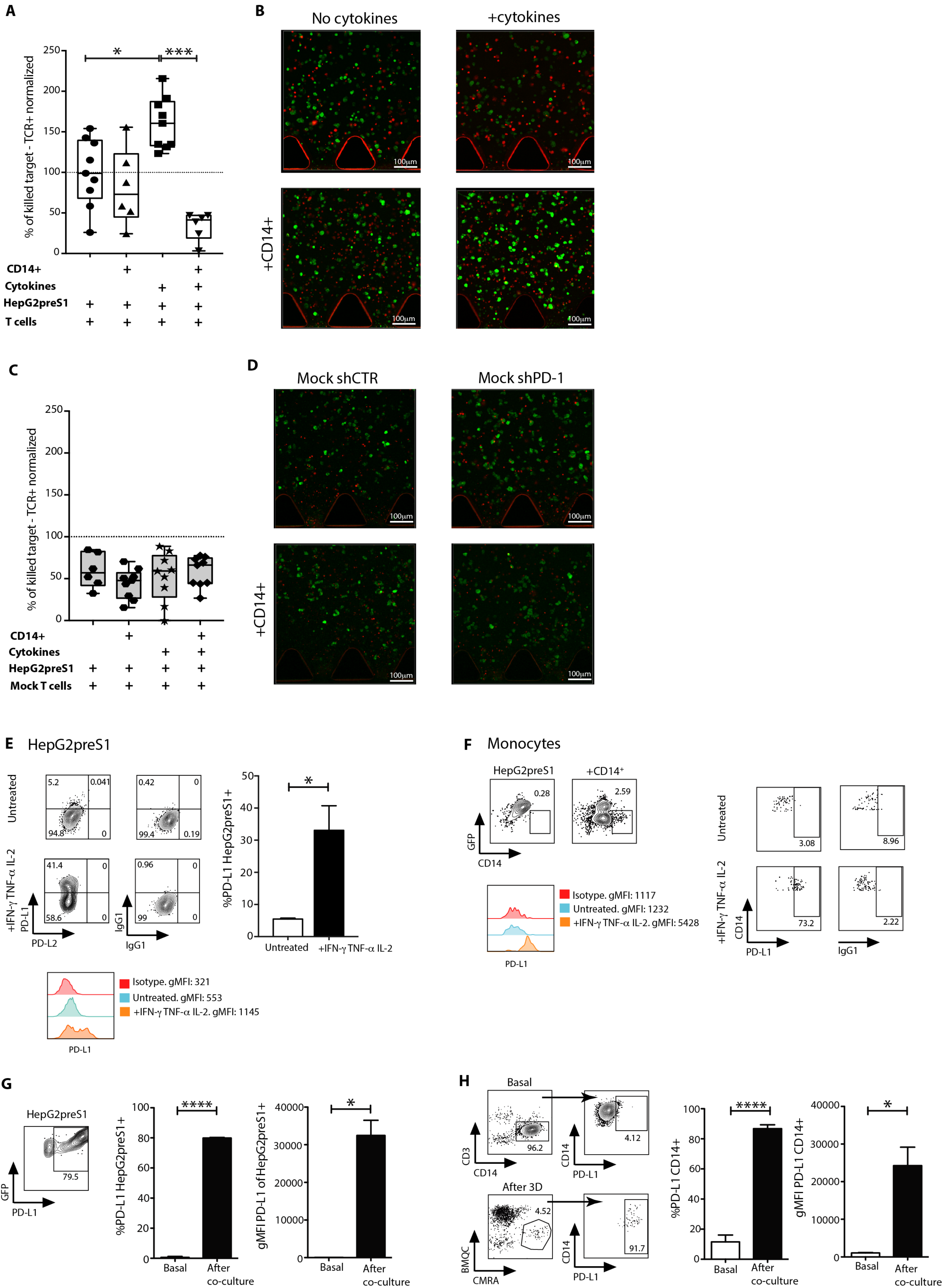

Supplementary Figure 4

A

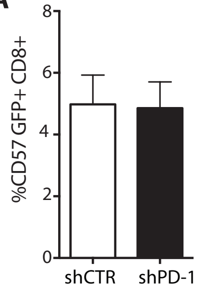

Supplement: Document S2. Article plus Supplemental Information [file mmc2.pdf]
